# Potential hepatoprotective effects of *Cistanche deserticola Y.C. Ma*: Integrated phytochemical analysis using UPLC-Q-TOF-MS/MS, target network analysis, and experimental assessment

**DOI:** 10.3389/fphar.2022.1018572

**Published:** 2022-10-12

**Authors:** Haichao Wang, Yaying Li, Yifei Bian, Xue Li, Yubei Wang, Ke Wu, Chuanguo Liu, Yuhong Liu, Xiaoming Wang

**Affiliations:** ^1^ College of Pharmaceutical Sciences, Shandong University of Traditional Chinese Medicine, Jinan, China; ^2^ Experimental Center, Shandong University of Traditional Chinese Medicine, Jinan, China; ^3^ Key Laboratory of Traditional Chinese Medicine Classical Theory, Ministry of Education, Shandong University of Traditional Chinese Medicine, Jinan, China; ^4^ Shandong Provincial Key Laboratory of Traditional Chinese Medicine for Basic Research, Shandong University of Traditional Chinese Medicine, Jinan, China; ^5^ Innovation Research Institute of Traditional Chinese Medicine, Shandong University of Traditional Chinese Medicine, Jinan, China

**Keywords:** PhGs, *Cistanche deserticola Y.C. Ma*, UPLC-Q/TOF-MS/MS, network analysis, hepatoprotective effect, TLR4/NF-κB signaling pathway

## Abstract

*Cistanche deserticola* Y.C. Ma (*CD*) possesses hepatoprotective activity, while the active ingredients and involved mechanisms have not been fully explored. The objective of this study was to investigate the chemical composition and hepatoprotective mechanisms of *CD*. We primarily used ultra-performance liquid chromatography with quadrupole time-of-flight tandem mass spectrometry (UPLC-Q-TOF-MS/MS) to identify the phenylethanoid glycoside (PhG) components of *CD*. Then, network analysis was used to correlate and predict the pharmacology of the identified active components of PhGs with hepatoprotection. Next, the mechanisms of the core components and targets of action were explored by cellular assays and toll-like receptor 4 (TLR4) target competition assays. Finally, its hepatoprotective effects were further validated in *in vivo* experiments. The results showed that a total of 34 PhGs were identified based on the UPLC-Q-TOF-MS/MS method. Echinacoside (ECH) was identified as the key ingredient, and TLR4 and nuclear factor-kappa B (NF-κB) were speculated as the core targets of the hepatoprotective effect of *CD via* network analysis. The cellular assays confirmed that PhGs had significant anti-inflammatory activity. In addition, the real-time quantitative polymerase chain reaction (RT-qPCR) and Western blot indicated that ECH notably reduced the levels of interleukin 6 (IL-6) and tumor necrosis factor alpha (TNF-α), as well as the mRNA expression of *TLR4*, *TNF-α*, and *IL-6*, and decreased the high expression of the TLR4 protein, which in turn downregulated the myeloid differentiation factor 88 (MyD88), p-P65 and TNF-α proteins in the inflammatory model. The target competition experiments suggested that ECH and LPS could competitively bind to the TLR4 receptor, thereby reducing the expression of TLR4 downstream proteins. The results of *in vivo* studies showed that ECH significantly ameliorated LPS-induced hepatic inflammatory infiltration and liver tissue damage and reduced serum alanine aminotransferase (ALT) and aspartate aminotransferase (AST) levels in mice. Moreover, ECH remarkably inhibited the release of inflammatory factors such as TNF-α, IL-6, IL-1β, and MCP-1 in the serum of mice, exerting the hepatoprotective effect by the TLR4/NF-κB signaling pathway. More importantly, ECH could act as a potential inhibitor of TLR4 and deserves further in-depth study. Our results could provide a basis for exploring the hepatoprotective properties of *CD*.

## 1 Introduction

The liver, the largest solid organ in the body, is a critical organ performing numerous physiological functions, including detoxification, immune system support, and metabolism and energy regulation (Kubes and Jenne, 2018; Xiao et al., 2019). However, various factors, such as obesity, cholestasis, viral hepatitis, alcohol, drug abuse, and lipopolysaccharide (LPS), can lead to liver diseases such as fatty liver, cirrhosis, hepatocellular carcinoma, and liver failure (Casey, 2016; Albillos et al., 2020; Burra, 2021). As a public health concern, liver disease is a major cause of morbidity and mortality all over the world, affecting millions of people, leading to approximately 2 million deaths every year worldwide. With the increase in liver-related deaths worldwide at present, liver disease led to significant public health problems and a huge medical burden (Hartke et al., 2017; Mundi et al., 2020; Huang et al., 2021; Zheng et al., 2021). The current treatment options for liver disease include lifestyle (dietary and activity) interventions, medical therapies, chemotherapy and targeted therapy agents, and liver transplantation, but overall benefits are modest (Price and Tien, 2017; Chalasani et al., 2018; Chitturi et al., 2018; Sim and Knox, 2018). Both their long-term effect and safety remain unknown, which limits their widespread use. Clearly, there exists an unmet need for innovative drugs. Fortunately, herbal medicine has clear advantages in the prevention and treatment of liver disease, showing good results and few side effects in clinical practice now (Zhang et al., 2018; Xu et al., 2020; Zhang et al., 2020; Xu G. et al., 2021).

*CD*, a parasitic plant in the mangosteen family, is globally distributed but mainly found in temperate zones. Also, it has been valued since ancient times as the “ginseng of the desert.” It was used as a homology of medicine and food and also has been extensively used in the food industry and clinical practice. As the major active components of *CD*, PhGs have been proven to have a wide range of pharmacological effects, such as antioxidant activity, anti-inflammation, anti-apoptosis, and immunological enhancement, and have been frequently prescribed for hepatoprotection (Li et al., 2016; Morikawa et al., 2019; Lei et al., 2020; Jiang et al., 2021). ECH is the major active component of PhGs isolated from *CD* (Liu et al., 2018a). Tao et al. (2021) found that ECH could prevent alcohol-induced liver injury and also had good hepatoprotective effects. Thida et al. (2021) found that ECH could improve the expression of hepatotoxicity by attenuating oxidative stress and inflammatory cytokines. In addition, isoacteoside is also one of the PhGs isolated from *CD* (Li et al., 2008)*.* Isoacteoside’s anti-inflammatory effect was found to be dependent on blocking TLR4 dimerization, which activated the MyD88-TAK1–NF-κB/MAPK signaling pathway cascades and the TRIF pathway, according to Gao et al, (2017). However, the mechanism underlying the therapeutic effects of *CD* in hepatoprotection has not been fully elucidated.

Recently, network analysis is widely applied in the study of herb medicine to explore the therapeutic targets and bioactive compounds (Wang et al., 2020a; Yin et al., 2021). As a “drug–target–disease” interaction network, network analysis helps assess the rationality and compatibility through constructing detailed compound–target and target–pathway networks (Guo et al., 2019; Zhang et al., 2019). It is a new research paradigm of drug discovery and has been proven to be effective in screening active components and potential targets in herbal medicine (Wang et al., 2020b; Song et al., 2020; Tian et al., 2020; Zhou et al., 2021; Montano et al., 2022). In addition, network analysis played an important role in screening targets of liver disease. For example, the treatment study of Erchen decoction in nonalcoholic fatty liver disease (NAFLD) has shown that the interactions of active ingredients of Erchen decoction with 77 targets related to NAFLD mainly reduces inflammation’s stimulation of the liver through the TLR4 signaling pathway (Liu et al., 2021). Xu et al. (2021b) used the network pharmacological method to analyze the mechanism of schisandrol B in the treatment of CCl4-induced liver injury. Therefore, we applied network analysis to screen new active components for hepatoprotection from PhGs of *CD* and predicted the potential mechanism of hepatoprotective activity. Finally, studies of the hepatoprotective activity of PhGs were carried out in *in vivo* and *in vitro* experiments.

**FIGURE 1:**
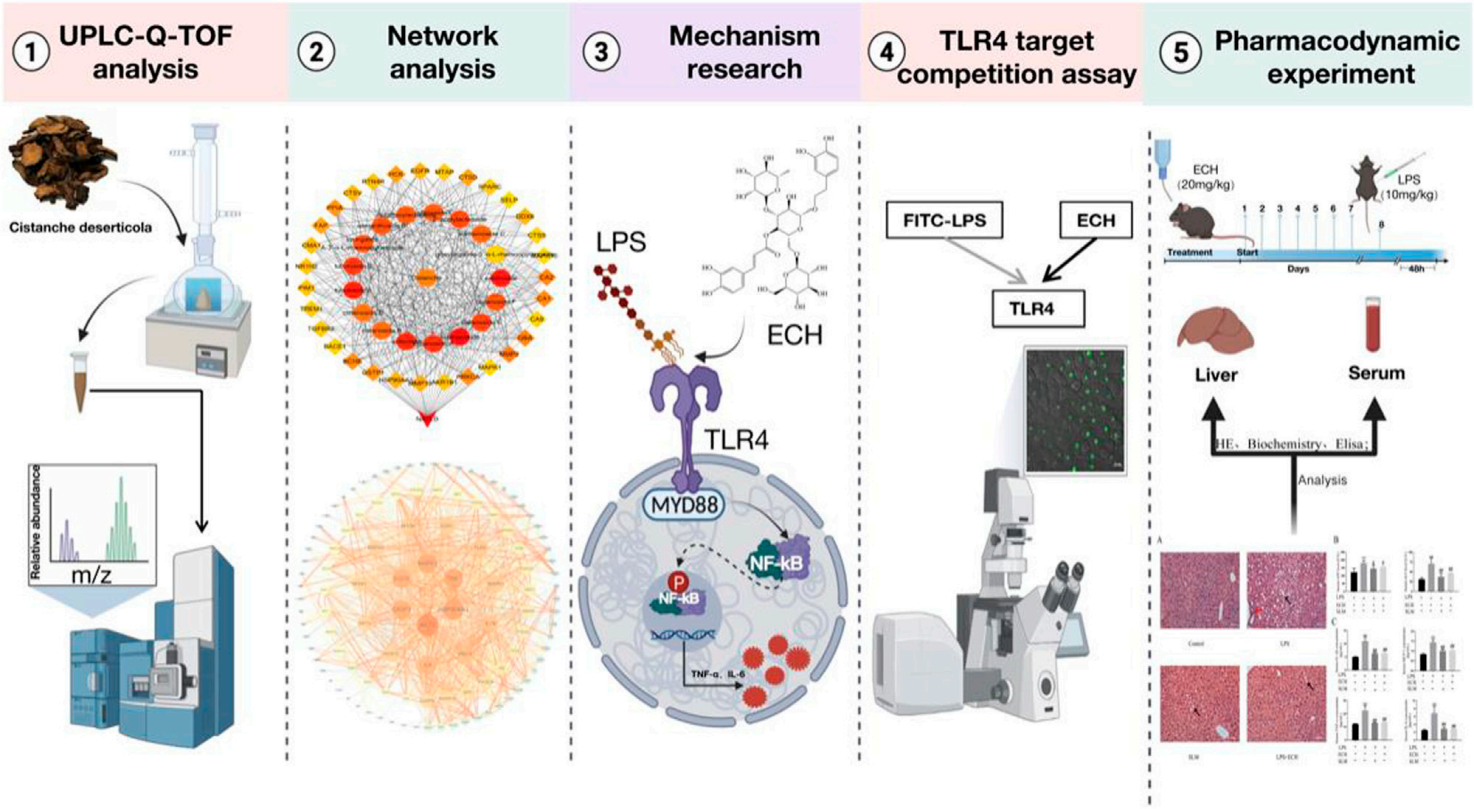
Comprehensive strategy diagram.

Using UPLC-Q/TOF-MS/MS technology combined with network analysis, we investigated the main components and potential targets of *CD* on hepatoprotection. The inflammatory model was established by inducing L02 cells with LPS, and the relevant pathways were studied through molecular biology and target competition experiments. Finally, the results from pharmacology were validated *in vivo*. In this study, we investigated the hepatoprotective effect and its potential mechanism of ECH so as to find a novel medicine for hepatoprotection from *CD*. The comprehensive strategy diagram is shown in Figure 1.

## 2 Materials and methods

### 2.1 Materials

CD was purchased from Ningxia province and identified by Professor Jun Chen from the Institute of Medicinal Plant Development. The reference standards of echinacoside, 2'-acetylacteoside, tubuloside A, acteoside, salidroside, and isoacteoside were purchased from Chengdu Munster Biotechnology Co., Ltd. (Chengdu, China); osmanthuside B, tubuloside B, cistanoside A, and poliumoside were supplied by Chengdu Keloma Biotechnology Co., Ltd. (Chengdu, China). The purities of all standards were above 98.0% as elucidated, and the details of the reference substances are given in Supplementary Table S1.

Thermo Fisher Scientific in the United States provided HPLC-grade methanol, acetonitrile, and formic acid. Watsons provided distilled water (Watsons Food and Beverage Co., Ltd., Guangzhou, China). The materials obtained are as follows: 1640 medium and fetal bovine serum (FBS) (Gibco, United States); penicillin–streptomycin liquid (Bioharp, Hefei, China); ECH (Chengdu Must Bio-technology Co., Ltd.; Chengdu, China); LPS (Meilunbio, Dalian, China); FITC-LPS (Sigma, United States); CCK-8 Kit and Simply P Total RNA Extraction Kit (Bioss, Beijing, China); MCP-1, IL-1β, IL-6, TNF-α, and NO Elisa Kits (Shanghai Enzyme-linked Biotechnology Co., Ltd., Shanghai, China); ALT and AST reagents (URIT Medical Electronic Co., Ltd., Guilin, China); StarLighter SYBR Green qPCR MIX and StarLighter Script RT all-in-one MIX (Beijing Foreverstar Biotech Co., Ltd., Beijing, China); PMSF, RIPA tissue/cell lysate (Beijing Solarbio Science and Technology Co., Ltd., Beijing, China); SDS-PAGE Sample Loading Buffer and BCA Protein Assay Kit (Beyotime Biotechnology Company, Beijing, China); PAGE Gel Fast Preparation Kit, Tris-glycine/SDS running buffer, transfer buffer, and TBST (Shanghai Epizyme Biomedical Technology Co., Ltd., Shanghai, China); primary antibody and secondary antibody dilution for Western blot (Absin, Shanghai, China). The detailed information of antibodies is shown in Supplementary Table S2.

### 2.2 Qualitative analysis of PhGs

#### 2.2.1 Preparation of the sample solution

The medicinal herbs (5 kg) underwent heated circumfluence extraction three times with 10 times the volume of 70% ethanol for 2 h each time, and the filtrates were combined and concentrated under reduced pressure to a thick paste. It was further purified by D101 macroporous resin and eluted sequentially with water and 50% ethanol. The fractions eluted with 50% ethanol were collected, concentrated, and dried under vacuum at 60°C to obtain the total glycoside fraction. The total glycoside (200 mg) was dissolved in 10 ml of 0.1% formic acid. After sonication, the sample solution was centrifuged at 12,000 rpm for 10 min, and the supernatant was subjected to LC–MS analysis.

#### 2.2.2 Preparation of the standard solution

Reference compounds were weighed separately to prepare the standard stock solution (1 mg/ml). An appropriate amount of the standard stock solution was taken and mixed, and then the mixed standard solution was analyzed by LC–MS.

#### 2.2.3 Establishment of the constituents’ database

To better identify compounds, a compound database of *CD* and its species was established by searching online databases, including TCMSP (http://lsp.nwu.edu.cn/tcmsp.php), PubMed (http://www.ncbi.nlm.nih.gov/pubmed), CNKI (http://www.cnki.net/), ChemSpider (http://www.chemspider.com/), *m/z* Cloud (https://www.mzcloud.org/), and PubChem (https://pubchem.ncbi.nlm.nih.gov/). Finally, 125 compounds, with their English name, molecular formula, molecular weight, and CAS numbers, were summarized.

#### 2.2.4 UPLC-Q-TOF/MS analysis conditions

The samples were separated on the Waters ACQUITY UPLC BEH C18 column (2.1 mm × 100 mm, 1.7 μm); the mobile phase (A) was acetonitrile, and the mobile phase (B) was water with 0.1% formic acid; the flow rate was 0.3 ml/min, the injection volume was 3 μL, and the column temperature was kept at 30°C. The gradient elution program is shown in Supplementary Table S3.

In the MS analysis, the data were obtained on the Synapt G2 high-definition mass spectrometer (HDMS) system (Waters Corporation, Milford, MA, United States) coupled with Q-TOF. The negative ion mode (NIM) has been used for electron spray ionization (ESI). Parameter settings: capillary voltage was 2.5 kV, ion source temperature was 120°C, the sampling cone was 10 V, the extraction cone was 3 V, desolvation temperature was 450°C, desolvation gas flow was 800 L/h, and cone gas flow was 40 L/h. Inter scan time was 0.02 s, scan time was 0.1 s, and scan range was *m/z* 50–1200; data format is centroid, and dynamic range is extended. 2 ng/L of leucine–enkephalin was used as real-time mass number correction (Lockspray mass: *m/z* 554.2615), and the frequency was 20 s. The MS data were collected and analyzed using MassLynx software v4.1.

#### 2.2.5 Identification strategies of chemical composition

**FIGURE 2:**
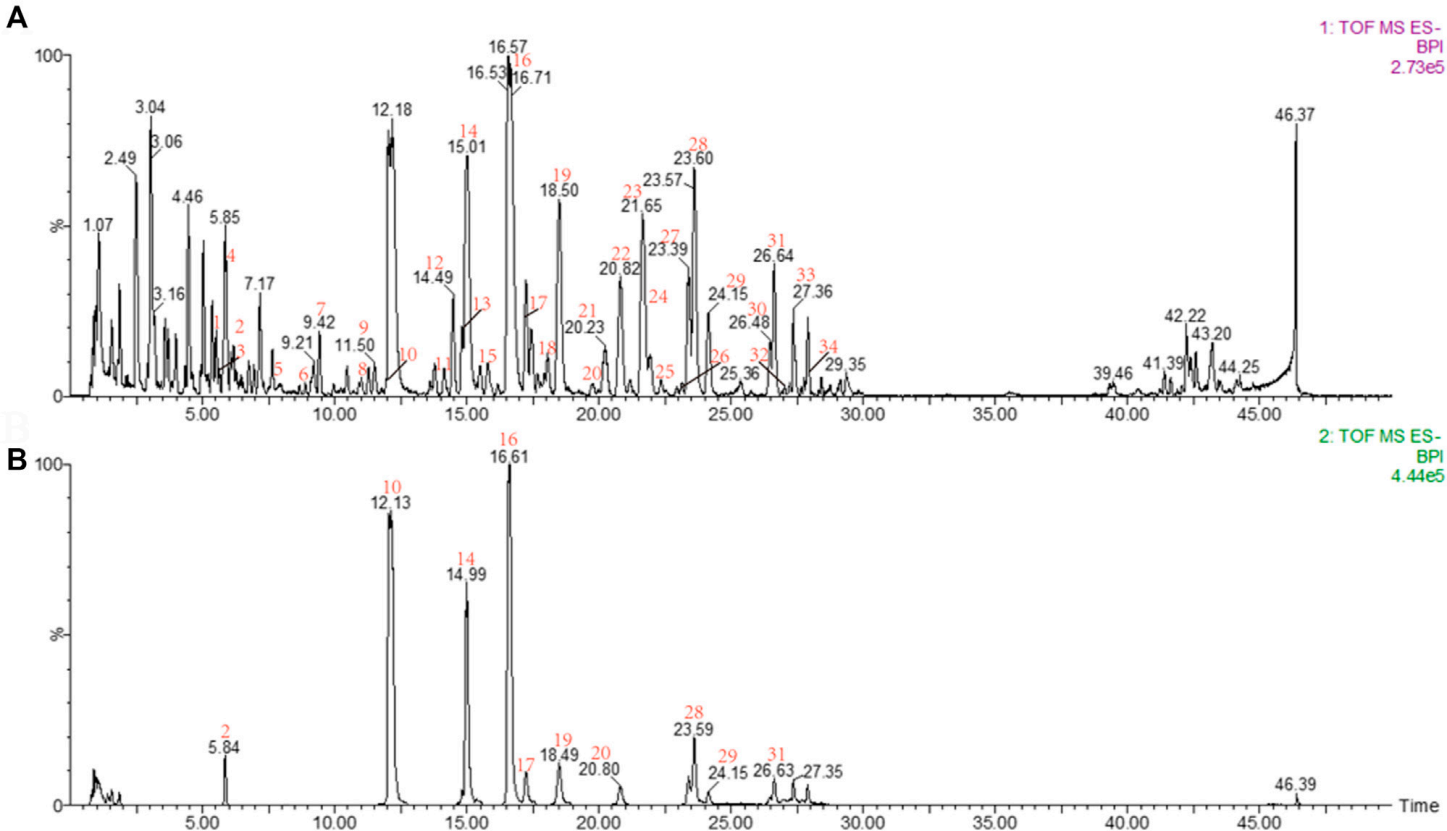
**(A)** BPI of the sample; **(B)** BPI of the reference substance: peak 2, salidroside; peak 10, echinacoside; peak 14, cistanoside A; peak 16, acteoside; peak 17, tubuloside A; peak 19, isoacteoside; peak 20, poliumoside; peak 28, 2′-acetylacteoside; peak 29, osmanthuside B; peak 31, tubuloside B.

Base peak intensity (BPI) chromatograms of *CD* acquired by UPLC-Q-TOF/MS are shown in Figure 2. First, the accurate mass of precursor ions was obtained by high-resolution mass spectrometry (HRMS). Second, the possible element composition (mass tolerance ≤ ± 5 ppm) of the compounds was speculated through MassLynx software v4.1, after which, referring to the double bond equivalents (DBE) and isotope fit (I-Fit), combined with the plant source, the most likely formula is matched with the established database, and the candidates are obtained according to the matching results.

The primary and multistage EICs were extracted and compared with the reference compounds and the MS^n^ reported in the literature. Also, peak attribution was determined by comparing the structures of the fragment ions with the candidates. For identification of isomers, this can be determined by comparing their retention times and MS^n^ with the reference substances.

### 2.3 Network analysis

#### 2.3.1 Prediction of *CD* candidate targets in NAFLD

To identify the targets, four databases were used to retrieve the chemical ingredients tested by UPLC-Q-TOF/MS: TCMSP (https://old.tcmsp-e.com/tcmsp.php), HERB (http://herb.ac.cn/), TCMIP (http://www.tcmip.cn/TCMIP/index.php/), and TCMID (http://www.megabionet.org/tcmid/). Also, SwissTargetPrediction, SEA, TCMSP, and HERB were used to predict the potential targets, where a probability greater than 0.75 is selected as the condition for predicting targets in the SwissTargetPrediction database. The following databases provided information on fatty liver disease-related targets: OMIM (https://www.omim.org/), TTD (http://db.idrblab.net/ttd/), and GeneCards (https://www.genecards.org/), after which, we screened the same targets of NAFLD and *CD*. Using Cytoscape v3.7.1 software, a “drug–target–disease” network was established.

#### 2.3.2 Candidate target functional enrichment and protein–protein interaction network analysis

To further understand the molecular activities of prospective targets, functional enrichment analysis was performed using the R package cluster profile. Then, the Kyoto Encyclopedia of Genes and Genomes (KEGG) pathways and Gene Ontology (GO) terms were enriched. In addition, the targets acted upon by the key constituents of *CD* obtained in network analysis were uploaded to the STRING database for protein–protein interaction (PPI) analysis to obtain the PPI network. Using the analysis tools in Cytoscape v3.7.1 software, the color size of the different nodes and the thickness of the lines between the nodes are defined according to the values of their properties.

#### 2.3.3 The molecular docking processing

The protein structures of the core targets were downloaded from the PDB database (http://www.rcsb.org/), separated from the water molecules using PyMOL 2.3.0 software, saved and imported into AutoDock Tools 1.5.6 software for hydrogenation, charge calculation, and atom type settings, and saved in a “pdbqt” format. The 3D structure of the active compound was drawn and energy minimization was performed using ChemOffice 18.0 software, saved, and imported into AutoDock Tools 1.5.6 in a “mol2” format for hydrogen addition and rotatable bond settings and then saved as a “pdbqt” format file. Molecular docking was performed using AutoDock Vina 1.1.2 software, and the ligand and receptor could bind well at binding energies less than -5 kJ/mol. Finally, the docking results were visualized using PyMOL 2.3.0 software.

### 2.4 Cell experiment

#### 2.4.1 Cell viability evaluation

The L02 cells were seeded in a 96-well plate overnight at 1×10^4^ cells/well and treated with PhGs (200, 100, 50, 25, and 0 μg/ml) and ECH (200, 100, 50, 25, and 0 μM). Following this incubation, the medium was aspirated, and the treated cells were cultivated for 90 min in the 1640 medium containing 10% CCK-8. An automated microplate reader was used to measure the absorbance at 450 nm.

#### 2.4.2 Enzyme-linked immunosorbent assay

The cells treated with drugs were collected, and the cell suspension was diluted with PBS (pH 7.2–7.4) to achieve a cell concentration of about 1 million/mL. Then, ultrasonic crushing was used to destroy cells and release cellular components, followed by centrifugation at 3000 rpm and 4°C for about 10 min. Also, the supernatant was carefully collected for detection. The content of the inflammatory factors (NO, TNF-α, and IL-6) was detected using ELISA kits in strict accordance with its instructions. First, 50 μL of the standard or sample was added to the appropriate wells, except for the blank well. Then, 100 μl of horseradish peroxidase (HRP) was added to standard wells and sample wells, except for the blank well, covered with an adhesive strip, and incubated for 60 min at 37°C. Next, after washing the microtiter plate five times, substrate A 50 μl and substrate B 50 μl were added to each well, following which, it was gently mixed and incubated for 15 min at 37°C in the dark. Finally, the stop solution was added, and a microplate reader set at a wavelength of 450 nm was used to measure the optical density (*OD*) value of each well in 15 min.

#### 2.4.3 RT-qPCR analysis

The Simply P Total RNA extraction kit was used to extract total RNA from the cells. Reverse transcription was conducted to obtain cDNA. Next, the cDNA was subjected to RT-qPCR instrumentation (Roche LightCycler 480) using amplification reagents. β-ACTB served as an endogenous control. The RT-qPCR primer sequences (Sewell, Wuhan, China) are shown in Supplementary Table S4.

#### 2.4.4 Cell culture and treatment

Beina Bio Co., Ltd (Beijing, China, BNCC35907) provided the L02 normal human liver cell line. L02 cells were cultured in the 1640 complete medium containing 10% FBS and 1% penicillin–streptomycin and maintained in a constant-temperature incubator at 37°C and 5% CO_2_. The control group and the LPS group were pretreated with a complete medium, but the ECH group was pretreated with 50 μM ECH. After being incubated for 24 h, both the LPS group and ECH group were cultured for 48 h in a complete medium containing 1 μg/ml LPS.

#### 2.4.5 Western blot

Treated cells were homogenized in RIPA buffer with PMSF and phosphatase inhibitors. The supernatant was obtained by sufficient centrifugation at 4°C (15 min, 14,000 rpm). The protein concentration was quantified with a BCA kit, and protein electrophoresis was performed on a 10% sodium dodecyl sulfate–polyacrylamide gel, following which the protein was transferred to a 0.45-μm PVDF membrane. Subsequently, PVDF membranes were immersed in milk containing 5% skim milk for 2 h at room temperature and incubated with the primary antibody overnight at 4°C. These membranes were washed three times with Tris-buffered saline containing 0.1% Tween 20, and then they were incubated with secondary antibodies for an additional 60 min at room temperature. Relative expression levels of proteins were obtained by delineating the expression levels of specific proteins using a β-actin reference band. Blots were detected using a GE Amersham Imager600 detection device. The experimental data were analyzed using ImageJ 1.8.0 software. Each experiment was repeated three times.

#### 2.4.6 Target competition assay

The cells were cultured in 20-mm confocal dishes and divided into four groups: the control group, the FITC-LPS group, the resatorvid+FITC-LPS group, and the ECH+FITC-LPS group. Only a complete medium was added to the control group. Only a complete medium containing 10 μg/ml of FITC-LPS was added to the FITC-LPS group. The resatorvid+FITC-LPS group was used as the positive control by adding the complete medium containing 150 μM TLR4 inhibitor and 10 μg/ml of FITC-LPS. To the ECH+FITC-LPS group was added the complete medium containing 200 μM of ECH solution and 10 μg/ml of FITC-LPS. After incubating the aforementioned four groups simultaneously for 90 min, the cell cultures were aspirated, discarded, rinsed 3–5 times using PBS, and then the phenol red free medium was added. Each group of experiments was repeated three times. Fluorescence detection was performed using a confocal fluorescence microscope (ZEISS LSM880+Fast Airyscan). The laser pinhole was opened at 100 nm, and the FITC green fluorescence channel was used with an excitation wavelength of 488 nm.

### 2.5 Animal experiment

#### 2.5.1 Animals and treatment

Male C57BL/6 mice (8-week-old; weight 16–20 g) were obtained from Weitonglihua Laboratory Animal Technology Co., Ltd. (Beijing, China). The animal experiments were carried out under the supervision of the Animal Experiment Center of Shandong University of Traditional Chinese Medicine, and the study protocol was approved by the Laboratory Animal Care and Use Committee of Shandong University of Traditional Chinese Medicine. The mice were housed in an (specified pathogen-free) SPF animal laboratory with standard temperature and humidity and subjected to a 12-h light–dark cycle. All animals had free access to food and water. To compare the efficacy of silymarin (SLM) as a positive drug with that of traditional ECH, all the mice were randomly divided into four groups (*n* = 12): the control group, the LPS group, the SLM+LPS group, and the ECH+LPS group. The LPS group, the SLM+LPS group, and the ECH+LPS group were given LPS at the same frequency at a dose of 10 mg/kg (i.p.), and the control group was given equal amounts of normal saline. To investigate the effect of ECH on LPS-induced liver injury, the mice in SLM and ECH groups were separately treated with SLM (36.4 mg/kg, i. g.) and ECH (20 mg/kg, i. g.) once on a daily basis for 7 days before LPS administration (Jia et al., 2014; Zhang et al., 2021). The control group and the LPS group were only given normal saline. After being given LPS 48 h later, the mice were anesthetized and dissected. Blood samples were collected, centrifuged at 3000 rpm for 15 min, and serum was separated for further analysis. Liver tissues were collected and quickly fixed in 4% paraformaldehyde or frozen in liquid nitrogen for further analysis.

#### 2.5.2 Histological evaluation of liver tissues

The liver samples were taken out from 4% paraformaldehyde, embedded in paraffin, and serially sectioned at 4-μm thickness. After routine deparaffinization, they were stained using hematoxylin and eosin (H&E). Histopathological examination was performed under a light microscope.

#### 2.5.3 Determination of biochemical indexes

Serum biochemical indexes, including alanine aminotransferase (ALT) and aspartate aminotransferase (AST), were detected by the automatic biochemical analyzer (URIT-8026; Guangxi, China).

#### 2.5.4 ELISA

The whole blood samples collected in the serum separation tube were placed at 4 °C overnight, centrifuged at 3000 rpm for 20 min, and then the supernatant was diluted twice in PBS for detection. The content of the inflammatory factors (TNF-α, IL-6, IL-1β, and MCP-1) was detected using ELISA kits in strict accordance with its instructions. The procedure is as described in Section 2.4.2.

### 2.6 Statistical analysis

Data are presented as the mean ± SD. The statistical analysis was carried out using SPSS 19.0 software (SPSS Inc, United States). One-sample t-tests were used to analyze the data after normalizing it to the control. Statistical significance is indicated as *p* < 0.05.

## 3 Results

### 3.1 Characterization of the chemical constituents

A total of 34 peaks were identified and determined to be PhGs according to the strategy in “Section 2.2.5" (Supplementary Figure S1). Ten components were validated against their reference compounds, and the chemical structures of the remaining peaks were inferred from the exact mass and MS^n^ fragment ions. The detailed information on ESI-MS and MS^n^ is shown in Table 1. The procedures of identification are as follows.

**TABLE 1:** Annotation and analysis of PhGs in *CD*.

No.	RT. (min)	[M−H]^–^(*m/z*)	Error (ppm)	Formula	MS/MS fragment ions (*m/z*)	Identification
1	5.52	623.2180	−1.1	C_26_H_39_O_17_	461.1651, 315.1106, 179.0349, 135.0449	Unknown
2	5.69	461.1662	0.7	C_20_H_29_O_12_	315.1082, 179.0349	Decaffeoylacteoside
3	5.77	299.1133	0.7	C_14_H_19_O_7_	119.0500	Salidroside^a^
4	6.07	487.1456	0.8	C_21_H_27_O_13_	179.0353, 161.0244, 135.0453	Cistanoside F
5	7.64	475.1813	−0.6	C_21_H_31_O_12_	179.0348, 135.0452	Cistanoside E
6	9.17	503.1761	−0.8	C_22_H_31_O_13_	461.1654, 315.1068, 179.0348, 161.0235	Unknown
7	9.42	801.2456	0.4	C_35_H_45_O_21_	783.2338, 639.2141, 461.1657, 179.0353,	Cistantubuloside C1/C2
8	11.28	639.1915	−1.6	C_29_H_35_O_16_	621.1799, 459.1486, 179.0345, 161.0243	Campneoside II or isomer
9	11.50	639.1929	0.6	C_29_H_35_O_16_	621.1825, 459.1520, 179.0349, 161.0246	Campneoside II or isomer
10	12.06	785.2492	−1.5	C_35_H_45_O_20_	623.2180, 477.1604, 315.1084, 161.0245	Echinacoside^a^
11	14.14	769.2548	−0.9	C_35_H_45_O_19_	607.2213, 461.1665, 179.0350, 161.0244	Cistantubuloside A
12	14.49	769.2541	−1.9	C_35_H_45_O_19_	623.2170, 477.1612, 163.0396, 153.0552	Cistantubuloside B1/B2
13	14.82	799.2654	−0.9	C_36_H_47_O_20_	785.2506, 623.2183, 461.1643, 193.0504,	Unknown
14	15.01	799.2674	1.6	C_36_H_47_O_20_	785.2513, 637.2351, 179.0354, 149.0607	Cistanoside A^a^
15	15.81	755.2387	−1.6	C_34_H_43_O_19_	593.2087, 551.1763, 447.1517, 195.0657	Unknown
16	16.58	623.1987	1.8	C_29_H_35_O_15_	461.1675, 315.1092, 179.0354, 161.0250	Acteoside^a^
17	17.24	827.2615	0.6	C_37_H_47_O_21_	665.2293, 623.2187, 477.1606, 161.0247	Tubuloside A^a^
18	18.06	813.2802	−1.8	C_37_H_49_O_20_	799.2632, 637.2350, 193.0506, 175.0397	Cistanoside B
19	18.50	623.1965	−1.8	C_29_H_35_O_15_	461.1652, 315.1070, 179.0346, 161.0242	Isoacteoside^a^
20	19.96	769.2549	−0.8	C_35_H_45_O_19_	623.1957, 461.1666, 179.0350, 161.0243	Poliumoside^a^
21	20.21	607.2036	1.5	C_29_H_35_O_14_	445.1711, 179.0354, 161.0246	Syringalide A-3’-α-L-rhamnopyranoside
22	20.82	607.2015	−2.0	C_29_H_35_O_14_	461.1642, 163.0392, 145.0289	Isosyringalide-3’-α-L-rhamnopyranoside or isomer
23	21.65	637.2129	−0.5	C_30_H_37_O_15_	623.2010, 475.1826, 329.1222, 161.0244	Cistanoside C
24	21.93	637.2130	−0.3	C_30_H_37_O_15_	623.1945, 461.1643, 193.0504, 175.0398	Plantainoside C or isomer
25	22.39	753.2605	−0.1	C_35_H_45_O_18_	179.0344, 161.0242, 135.0447	Kankanoside I
26	23.16	607.2015	−2.0	C_29_H_35_O_14_	461.1659, 163.0391, 145.0292	Isosyringalide-3’-α-L-rhamnopyranoside or isomer
27	23.39	637.2131	−0.2	C_30_H_38_O_15_	623.1999, 461.1659, 193.0508, 175.0391	Plantainoside C or isomer
28	23.60	665.2087	0.8	C_31_H_37_O_16_	623.1995, 503.1774, 461.1662, 179.0349	2’-Acetylacteoside^a^
29	24.15	591.2087	1.5	C_29_H_35_O_13_	445.1680, 163.0405, 145.0290	Osmanthuside B^a^
30	26.46	651.2292	0.5	C_31_H_39_O_15_	475.1805, 193.0660, 175.0399, 149.0602	Cistanoside D
31	26.64	665.2067	−2.3	C_31_H_37_O_16_	623.1964, 503.1755, 461.1658, 179.0345	Tubuloside B^a^
32	27.02	649.2117	−2.3	C_31_H_37_O_15_	607.2004, 503.1757, 163.0398, 145.0293	Salsaside F
33	27.35	679.2233	−0.7	C_32_H_39_O_16_	637.2119, 623.1975, 179.0347, 161.0244	Cistansinenside A or salsaside E
34	27.91	679.2232	−0.9	C_32_H_39_O_16_	637.2107, 623.2013, 179.0347, 161.0244	Cistansinenside A or salsaside E

“a” represented the compound confirmed by comparison with the reference standards.

### 3.1.1 MS cleavage behaviors

In the NIM, the PhGs exhibited similar fragmentation patterns, mainly involving the loss of some characteristic neutral fragments, such as caffeoyl (CA, C_9_H_6_O_3_, 162 Da), feruloyl (Fr, C_10_H_8_O_3_, 174 Da), coumaroyl (Cm, C_9_H_6_O_2_, 146 Da), acetyl (Ac, C_2_H_2_O, 42 Da), glucose (Glu, C_6_H_10_O_5_, 162 Da), rhamnose (Rha, C_6_H_10_O_4_, 146 Da), and dehydration (18 Da).

The compositions of PhGs can be quickly identified based on the characteristic fragment ions. In addition to the aforementioned characteristic neutral loss, a series of fragment ions were their moieties (including glycosides and aglycone) in the low *m/z* range. For example, the peak of the fragment ion at *m/z* 179.03 was from caffeoyl, and the peaks of fragment ions at *m/z* 161.02 and *m/z* 135.04 were obtained from the removal of H_2_O and CO_2_; the peak of the fragment ion from the aglycone (hydroxytyrosol) at *m/z* 153.06 and that at *m/z* 123.04 were generated by the removal of CH_2_O.

In this experiment, the fragmentation pathways of reference compounds (Table 1) were analyzed using UPLC-Q-TOF/MS. The characteristic diagnostic ions related to their structures were summarized, and their possible fragmentation pathways were speculated based on the multilevel mass spectra information of the reference substances. The aforementioned results are shown in Supplementary Figure S2.

### 3.1.2 Identification of PhGs

The relevant literature shows that *CD* contains a large number of PhGs, and the chemical structures of these components are generally glycosides of the phenylethanol group or phenylethanol group with hydroxyl/methoxy substitution attached to β-D-glucopyranose/β-D-glucopyranoside with substituents at the C-2∼6 position (Jiang et al., 2009; Han et al., 2012; Holzgrabe and Malet-Martino, 2014). The structural features of PhGs are summarized as follows: the benzene ring is substituted with hydroxyl or methoxy at the C-3 and C-4 positions in the glycosides. Phenylpropenylation often occurs at the C-4 or C-6 position of the medial glucose, and the types of phenylpropenyl groups include caffeoyl, coumaroyl, and feruloyl. Also, the two alkene hydrogens on acryloyl are mostly trans-structures, with occasional acetylation at the C-2 position of the central sugar, and these moieties are linked by ester or glycosidic linkages, which are prone to form the corresponding fragment ions during in-source fragmentation.

**FIGURE 3:**
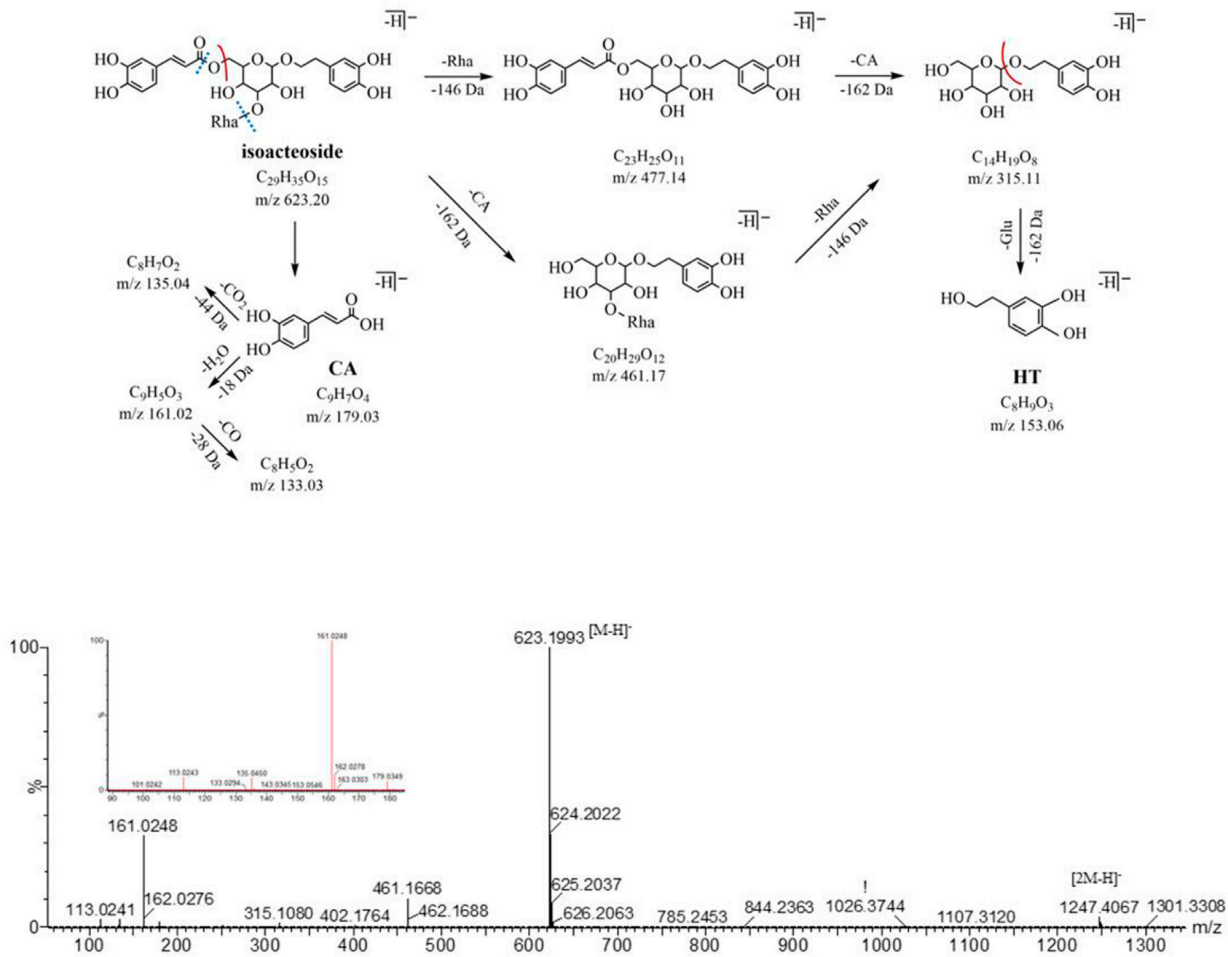
MS^2^ and the fragmentation pathway of the isoacteoside.

In the NIM, both peak 16 and peak 19 (eluted at 16.58 and 18.50 min, respectively) are quasi-molecular ions at *m/z* 623.20 [M-H]^-^ with a formula of C_29_H_35_O_15_, for a pair of isomers, could produce the similar fragment ions *m/z* 461.17, 315.11, 179.03, 161.02, and 135.05 in the MS^2^ spectrum. The neutral loss of caffeoyl (C_9_H_6_O_3_, -162 Da) forms fragment ions *m/z* 461.17 (C_20_H_29_O_12_) by ester bond cleavage, which further loses a neutral molecule of rhamnose (C_6_H_10_O_4_, -146 Da) and produces the ion *m/z* 315.11 (C_14_H_19_O_8_). In the low range of *m/z*, we found the characteristic fragment ions associated with the caffeoyl structure (*m/z* of 179, 161, and 135) and aglycone structure (*m/z* 153.06 and 123.04). After searching the compound library, acteoside and isoacteoside were matched to them. Peaks 16 and 19 were identified as acteoside and isoacteoside, respectively, by comparing their MS^2^ fragmentation pathways and retention times with the reference compounds, and their MS^2^ spectrum and fragmentation pathway are shown in Figure 3.

**FIGURE 4:**
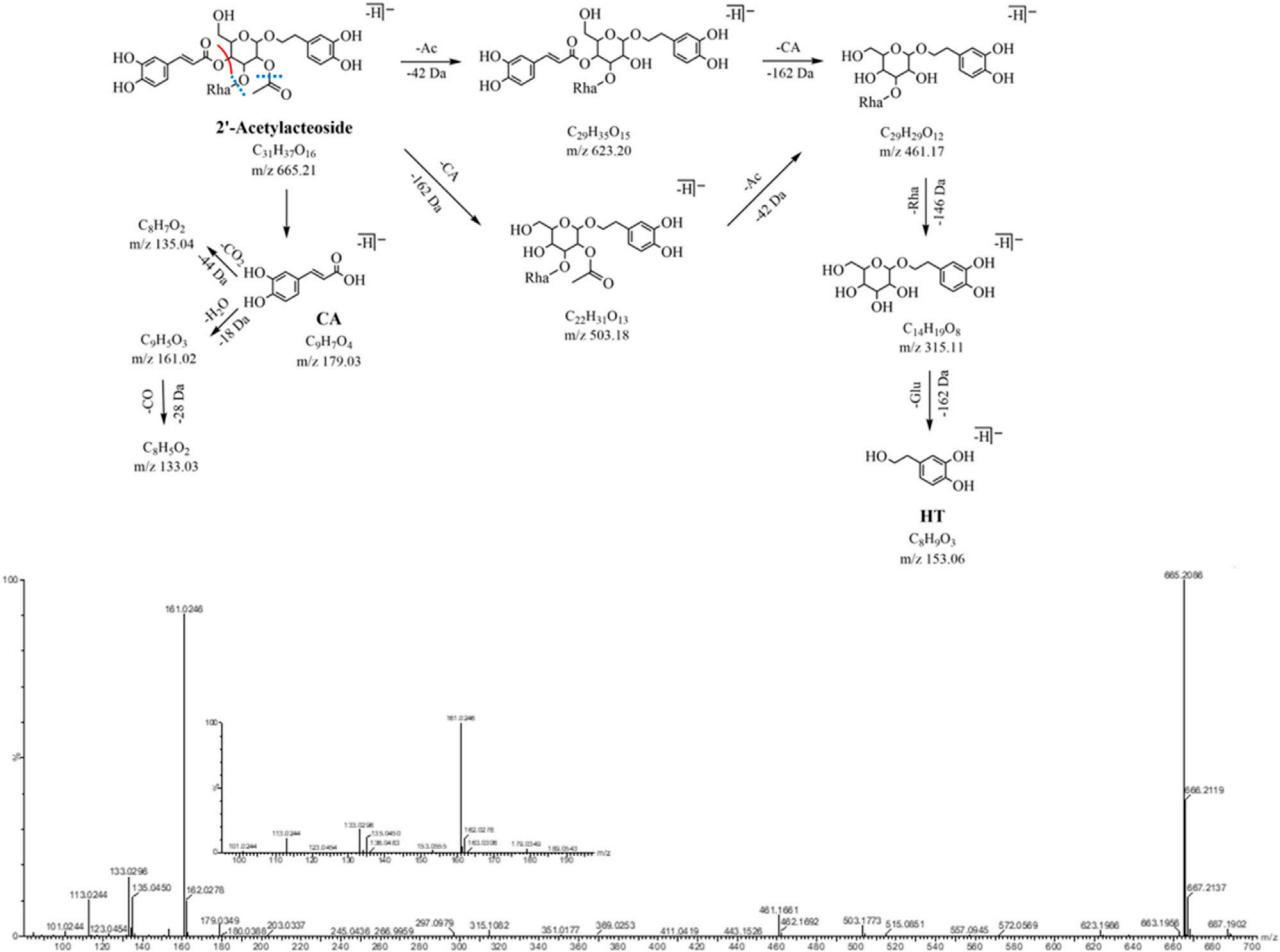
MS^2^ and the fragmentation pathway of the 2’-acetylacteoside.

The quasi-molecular ion peaks for both peak 28 and peak 31 (*m/z* 665.21, C_31_H_37_O_16_, eluted at 23.60 and 26.64 min, respectively) are at *m/z* 665.21. Compared with peaks 16 and 19, the added mass of 42 Da (C_2_H_2_O) indicated that peak 28 and peak 31 were both present at the 2-hydroxyl acetylation position of the central sugar (glucose). The neutral loss of the acetyl group (C_2_H_2_O, -42 Da) in the MS^2^ spectrum forms a corresponding fragment ion *m/z* 623.20 (C_29_H_35_O_15_). The fragment ion *m/z* 503.18 (C_22_H_31_O_13_) attributed to the loss of neutral (C_9_H_6_O_3_, -162 Da) and other identical fragment ions *m/z* 461.17 and *m/z* 315.11. The characteristic fragmentation ions *m/z* 179.03, 161.02, and 135.04 related to the caffeoyl structure and the feature fragment ions at *m/z* 153.06 and 123.04 related to the aglycone structure were observed. Peaks 28 and 31 were identified as 2'-acetylacteoside and tubuloside B, respectively, by comparing their MS^2^ cleavage behaviors and retention times with those of the reference compounds. The MS^2^ profile and fragmentation pathway are shown in Figure 4.

**FIGURE 5:**
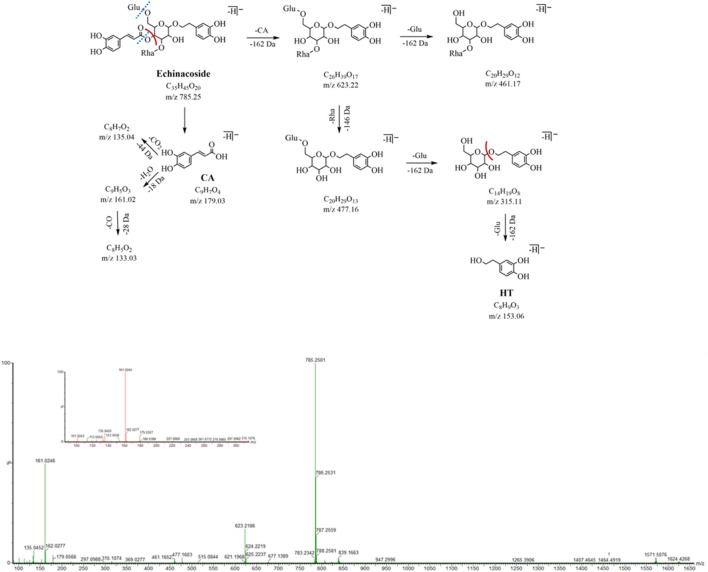
MS^2^ and the fragmentation pathway of the ECH.

Peak 10 (*m/z* 785.25, C_35_H_45_O_20_, 12.06 min) has 162 Da (Glu, C_6_H_10_O_5_) more than peak 16 and peak 19 (*m/z* 623.20, C_29_H_35_O_15_), a glucose glycosyl group, which is presumed to be a triglycoside with another molecule of glucose attached to the central sugar (glucose). The neutral loss of caffeoyl (C_9_H_6_O_3_, -162 Da) in the MS^2^ spectrum forms fragment ions at *m/z* 623.22 (C_26_H_39_O_17_), which further loses the neutral molecules of rhamnose (C_6_H_10_O_4_, 146 Da) and glucose (C_6_H_10_O_5_, 162 Da) and yields the corresponding fragment ions *m/z* 477.16 (C_20_H_29_O_13_) and *m/z* 461.17 (C_20_H_29_O_12_). The characteristic fragmentation ions at *m/z* 179.03, 161.02, and 135.04 related to the caffeoyl structure as well as the feature fragment ions at *m/z* 153.06 and 123.04 related to the aglycone structure were still observed. Peak 10 was identified as ECH by comparing its MS^2^ cleavage behavior and retention time with those of the reference compound, and its MS^2^ spectrum and cleavage pathway is shown in Figure 5.

**FIGURE 6:**
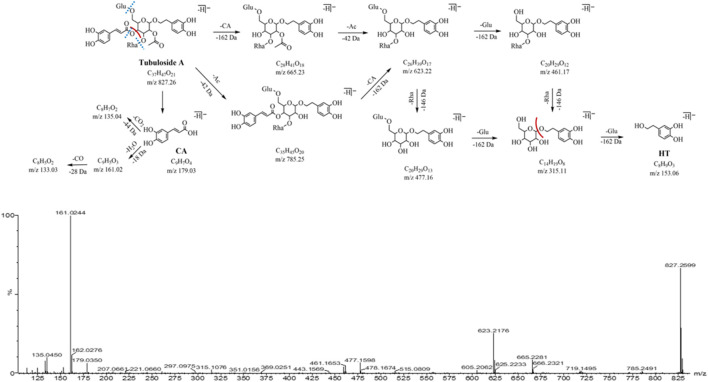
MS^2^ and the fragmentation pathway of the tubuloside A.

Peak 17 (*m/z* 827.26, C_37_H_47_O_21_, 17.24 min) has 42 Da (C_2_H_2_O, an acetyl group) more than peak 10 (*m/z* 785.25, C_35_H_45_O_20_), presuming that peak 17 is a triglycoside with hydroxyacetylation at the 2-position of the central sugar (glucose). The neutral loss of caffeoyl (C_9_H_6_O_3_, -162 Da) in the MS^2^ spectrum forms a fragment ion *m/z* 665.23 (C_28_H_41_O_18_), which further loses acetyl (C_2_H_2_O, -42 Da) and yields the same fragment ions *m/z* 623.22, 477.16, and 461.17 in the peak 10. The characteristic fragment ions *m/z* 179.03, 161.02, and 135.04 related to the caffeoyl structure were observed, as well as the characteristic fragment ions *m/z* 153.06 and 123.04 related to the aglycone structure. Peak 17 was identified as tubuloside A by comparing their MS^2^ cleavage behavior and retention time with those of the reference compounds. The MS^2^ spectrum and fragmentation pathway is shown in Figure 6.

The fragmentation patterns of several other typical compounds are described in Supplementary Material S1.

## 3.2 Results of network analysis

### 3.2.1 Screening the targets

**FIGURE 7:**
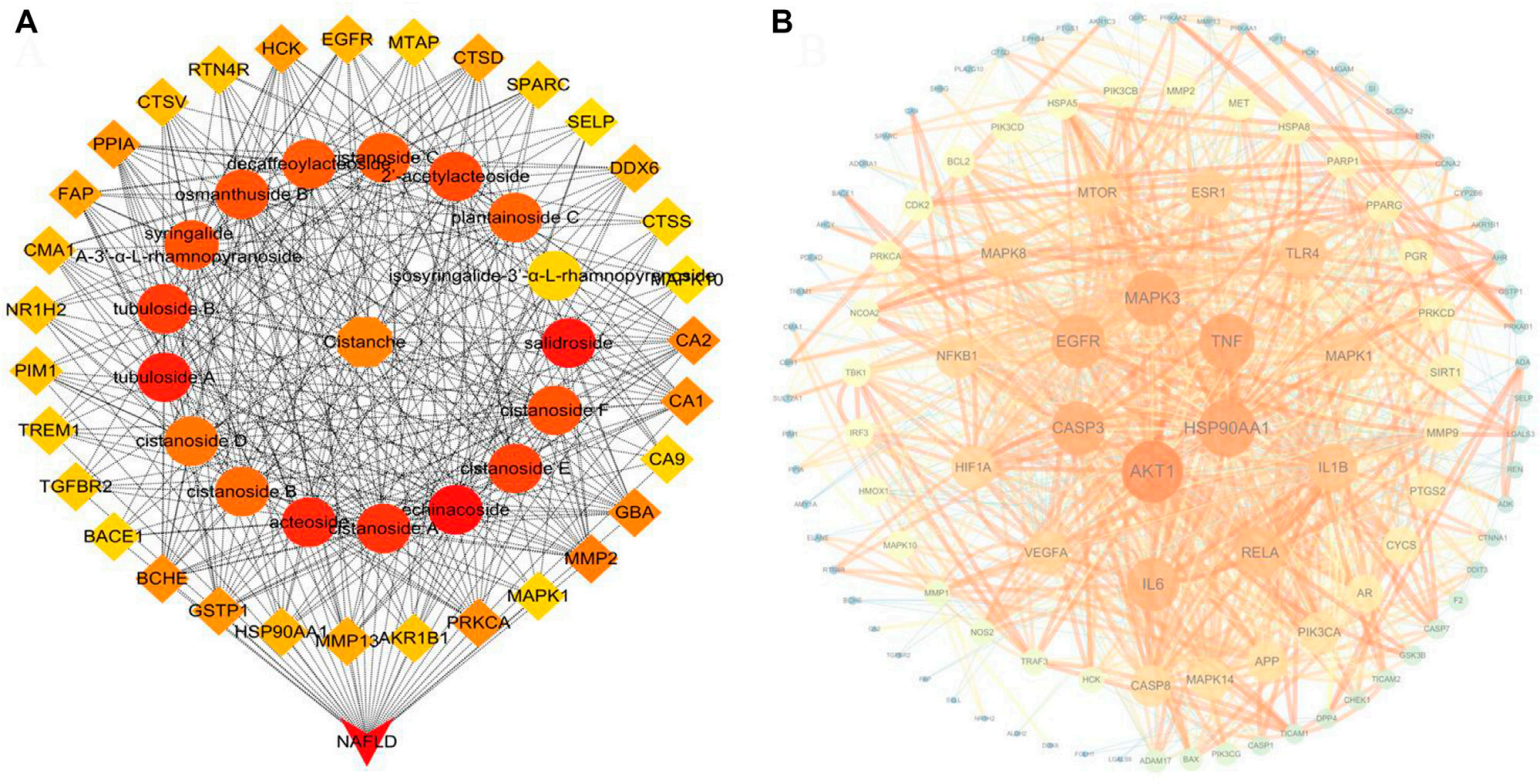
**(A)** Top 50 nodes of the compound–drug–target–disease network; **(B)** PPI network of *CD* candidate targets.

We further combined the identified 34 compounds with the database of traditional Chinese medicine ingredients to conduct an active compound search and screening to obtain 17 active ingredients. Finally, 189 targets corresponding to these 17 compounds were found in the online database. Also, 1963 NAFLD-related targets were acquired using OMIM, GeneCards, and TTD resources. We screened 135 overlapping targets as prospective targets linked to NAFLD by intersecting the targets of compounds with NAFLD-related targets (Supplementary Figure S3). To illustrate the drug–target–disease network, 135 possible targets and 17 compounds were loaded into Cytoscape 3.7.1 software. In this network, echinacoside, salidroside, and acteoside ranked as the top three compounds, connecting the largest number of targets (Figure 7A). In addition, 15 compounds were bound to the TLR4 target, and the predicted results showed that ECH had the most stable binding effect on the TLR4 protein. The experimental results are shown in Supplementary Table S5.

#### 3.2.2 The PPI network and functional enrichment of candidate targets

**FIGURE 8:**
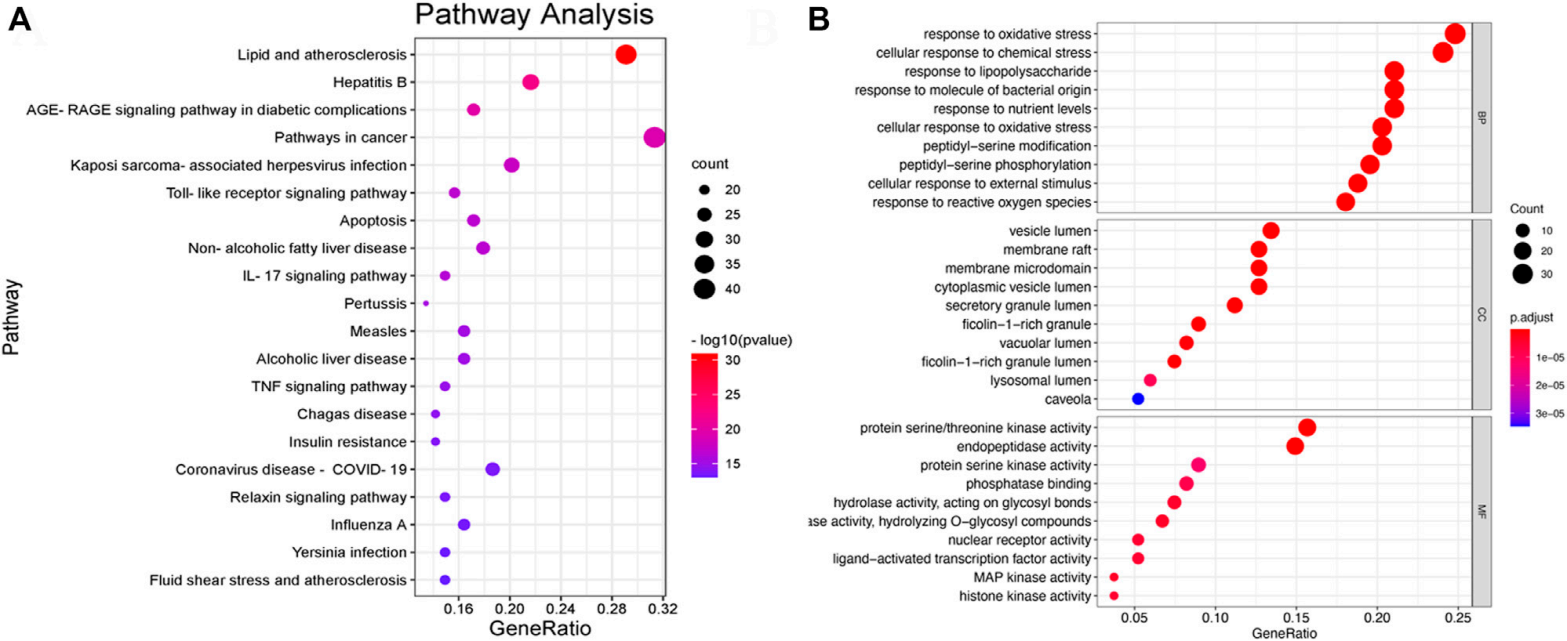
**(A)** Bar plot of KEGG pathway enrichment analysis results, showing the top 20 pathways in the KEGG. **(B)** Bar plot of GO pathway enrichment analysis results, showing the top 10 pathways in the GO.

We performed PPI network and functional enrichment analyses to reveal interactions between candidate target genes. Also, their associated biological functions and metabolic pathways were investigated. As shown in Figure 7B, the higher the ranking, the larger the circle and the darker the color, indicating higher relevance. AKT1, HSP90AA1, and TNF have been discovered to play key roles in the PPI network. The functions in the KEGG and GO analyses are shown in Figures 8A,B. The results of KEGG pathway enrichment analysis were largely associated with lipid and atherosclerosis, hepatitis B, the AGE-RAGE signaling pathway in diabetic complications, pathways in cancer, Kaposi sarcoma-associated herpesvirus infection, TLR signaling pathway, and so on. The GO biological process was closely related to the response to LPS and so on.

## 3.3 Results of cell experiments

### 3.3.1 Optimal intervention condition on L02 cells was determined

**FIGURE 9:**
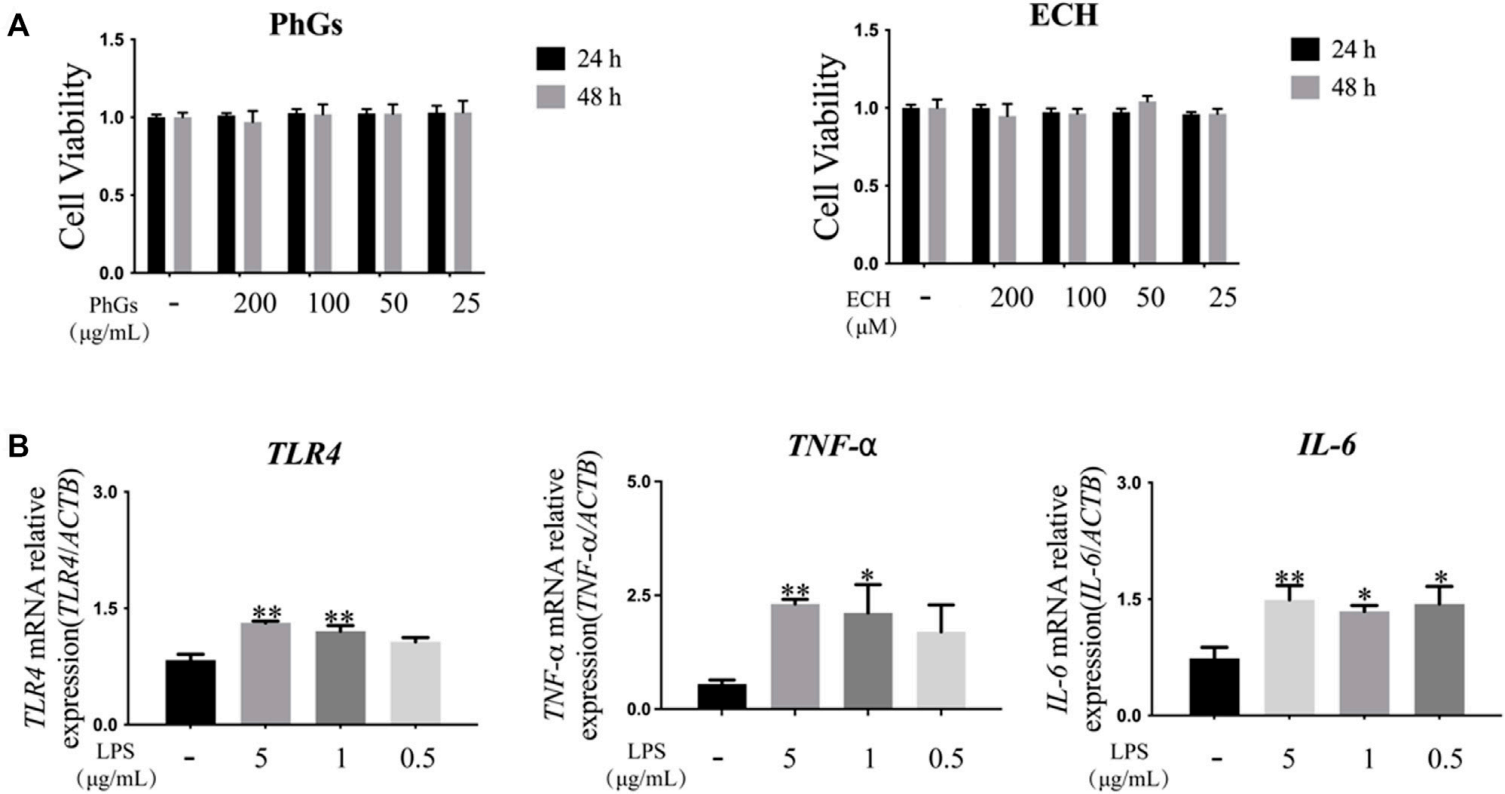
Establishment of the inflammation model in L02 cells. **(A)** Cell viability of L02 was affected by different doses and duration of action of PhGs and ECH (*n* > 3). **(B)** Expression of *TLR4*, *IL-6*, and *TNF-α* genes in L02 cells cultured with different concentrations of LPS (*n* = 3). *p < 0.05 and **p < 0.01 control group compared with the LPS group; all data are presented as mean ± SD.

To assess the cytotoxicity of PhGs and ECH on L02 cells, CCK-8 assays were performed. The results in Figure 9A indicated that the concentration of PhGs in the range of 25–200 μg/ml and that of ECH in the range of 25–200 μM had no toxic effect on cells. In addition, the effects of LPS on L02 cells are shown in Supplementary Figure S4. In the next step, we treated L02 cells with LPS to establish a cellular inflammation model and performed an RT-qPCR assay. The RT-qPCR assay results showed that with respect to the control group, the mRNA expression of *IL-6*, *TLR4*, and *TNF-α* was increased in the LPS group. When choosing an intervention condition, an LPS concentration with the lowest concentration and the best effect should be chosen. Although the lowest concentration was found in the 0.5-µg/ml LPS group, it had an effect only on the mRNA expression of *IL-6* (*p* < 0.05). However, the 1-μg/ml LPS group showed a significant increase in the mRNA expression of *TLR4* (*p* < 0.01), *TNF-α* (*p* < 0.05), and *IL-6* (*p* < 0.05). Therefore, 1 μg/ml (LPS) was chosen for further study (Figure 9B).

### 3.3.2 PhGs inhibit the production of NO

**FIGURE 10:**
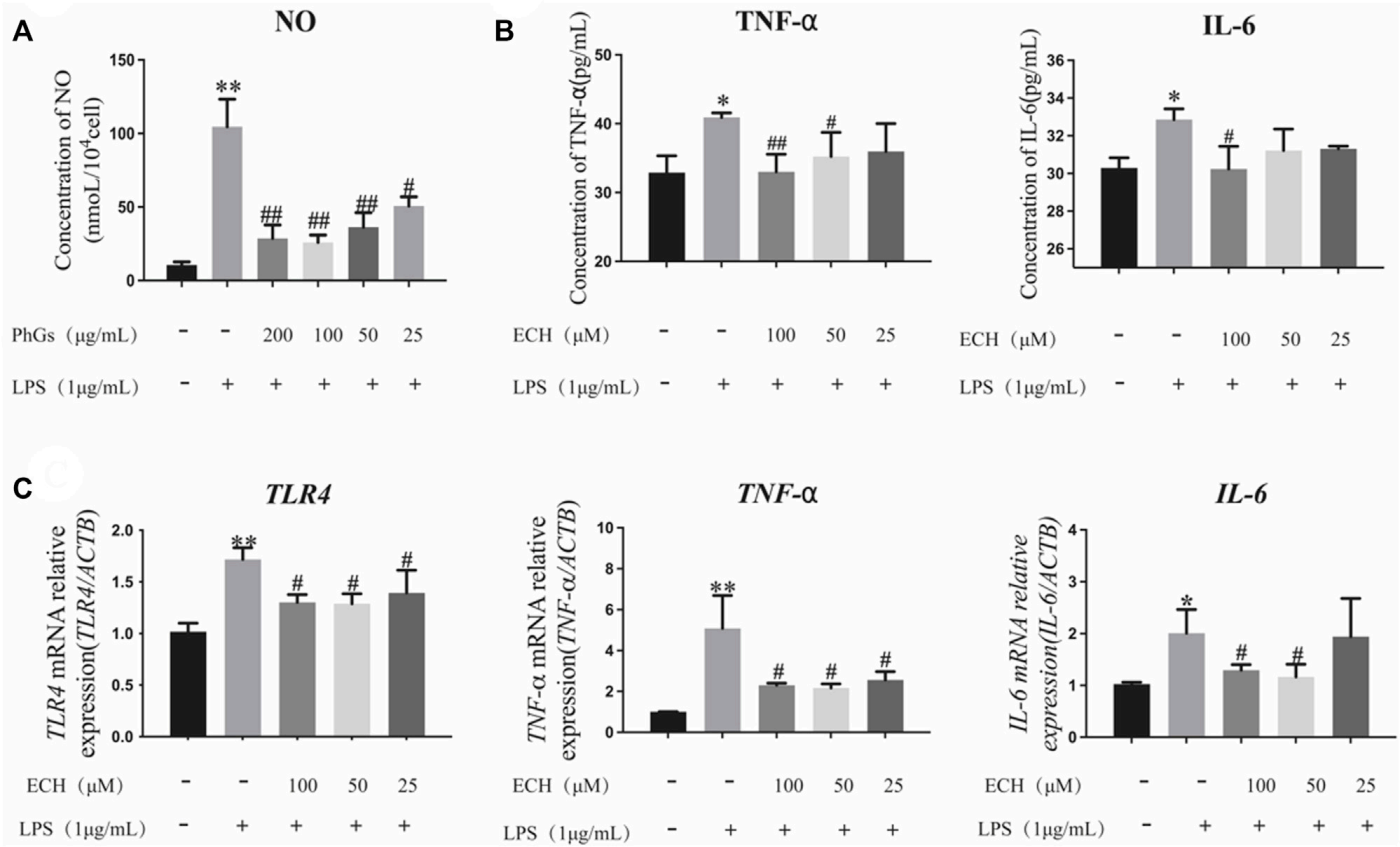
Effects of PhGs and ECH on inflammatory factors in L02 cells. **(A)** Expression of NO was analyzed by ELISA. **(B)** IL-6 and TNF-α expression assessed by ELISA. **(C)** Inflammation-related genes *TLR4*, *TNF-α*, and *IL-6* were tested by RT-qPCR; *p < 0.05 and **p < 0.01 compared with the control group; ^#^ *p* < 0.05 and ^##^ *p* < 0.01 compared with the LPS group; all data are presented as mean ± SD (*n* = 3).

Echinacoside is the indicative component for the determination of PhGs and is mainly used for liver protection, immune protection, etc. (Pei et al., 2018). Inflammation is closely related to oxidative stress, and with the aim of determining whether PhGs could inhibit LPS-induced inflammation in L02 cells, the level of NO was detected. Our results indicated that PhGs obviously decreased NO levels. 25-μg/mL PhG treatment significantly reduced NO production compared to the LPS group (*p* < 0.05). NO levels were reduced more significantly in the 200, 100, and 50 μg/ml PhG groups (*p* < 0.01, Figure 10A).

#### 3.3.3 ECH inhibits the production of pro-inflammatory cytokines

We measured the secretion of TNF-α and IL-6 using ELISA kits (Figure 10B). ECH administration reduced IL-6 and TNF-α production compared to the LPS group (*p* < 0.05). Among them, 100 μg/ml of ECH administration remarkably decreased the production of IL-6 (*p* < 0.05). TNF-α levels were significantly reduced in the 50-μg/ml ECH administration group (*p* < 0.05), and the 100-μg/ml ECH administration group had a more significant reduction in TNF-α levels (*p* < 0.01).

### 3.3.4 Effect of ECH on inflammatory gene expression

To determine the expression of inflammation-related genes in L02 cells induced by LPS with different concentrations of ECH, RT-qPCR analyses were performed. As shown in Figure 10C, LPS upregulated the levels of *TLR4*, *TNF-α*, and *IL-6* mRNA in L02 cells (*p* < 0.05), but ECH downregulated these increased mRNA levels. Among them, the high-concentration (100 μg/ml) and medium-concentration (50 μg/ml) administration groups were closer to the control group. These findings suggested that ECH inhibited LPS-induced inflammation in L02 cells.

#### 3.3.5 ECH inhibits the TLR4/NF-κB signaling pathway

**FIGURE 11:**
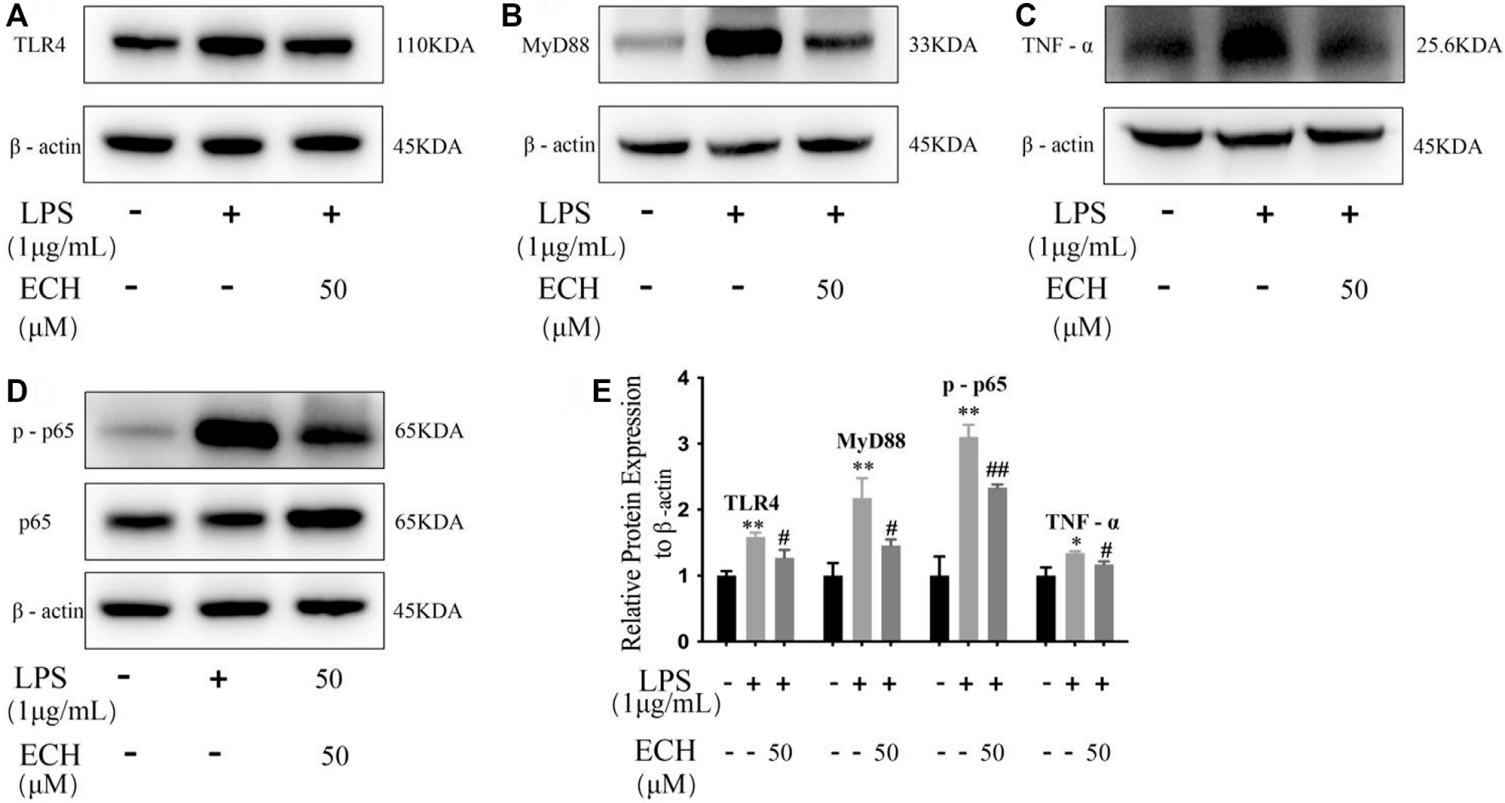
Influence of ECH on inflammatory response during the TLR4/NF-κB signaling pathway in L02 cells. **(A)** Protein expressions of TLR4. **(B)** Protein expressions of MyD88. **(C)** Protein expressions of TNF-α. **(D)** Protein expressions of p-P65; **(E)** Protein expression levels of TLR4, MyD88, p-65, and TNF-α the control group compared with the LPS group *p < 0.05 and **p < 0.01; ECH group compared with the LPS group ^#^ *p* < 0.05 and ^##^ *p* < 0.01; all data are presented as mean ± SD (*n* = 3).

To investigate the anti-inflammatory effect of ECH, we detected the expression of TLR4, MyD88, p-P65, and TNF-α to identify whether it regulated inflammation through the TLR4/NF-κB signaling pathway. ECH treatment significantly reduced the TLR4 protein compared to the LPS group (*p* < 0.05). At the same time, ECH also decreased the expression of its downstream proteins MyD88 (*p* < 0.05) and p-P65 (*p* < 0.01). Similarly, ECH decreased TNF-α protein levels in LPS-treated L02 cells (*p* < 0.05). These results indicated that ECH may suppress inflammation by adjusting the TLR4/NF-κB signaling pathway (Figure 11).

#### 3.3.6 ECH competes with FITC-LPS for cellular receptor binding sites

**FIGURE 12:**
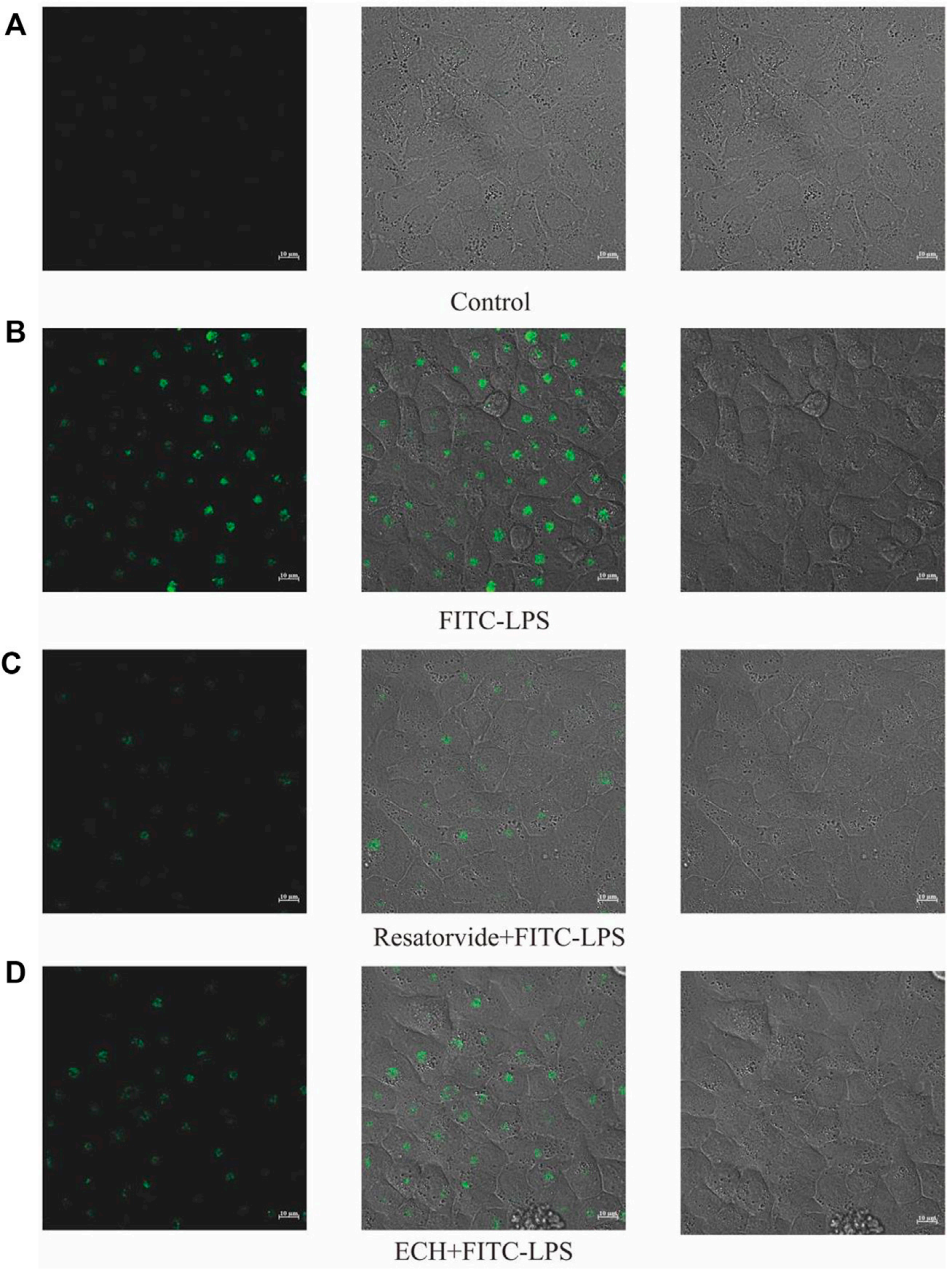
Effects of ECH on competitive inhibition of FITC-LPS binding cells. **(A)** Control group; **(B)** FITC-LPS group; **(C)** resatorvide+FITC-LPS group (TLR4 antagonist); **(D)** ECH+FITC-LPS group.

The binding of FITC-LPS to cell membrane surface receptors resulted in enhanced fluorescence at the cell membrane surface, but co-incubation of the compound with FITC-LPS competitively inhibited the binding of FITC-LPS to the membrane surface receptors, resulting in diminished fluorescence intensity (Troelstra et al., 1997; Sauter et al., 2007). As shown in Figure 12, the fluorescence intensity of the FITC-LPS group was the highest after the binding of LPS with fluorescent labeling to cells. When the TLR4 inhibitor was incubated with FITC-LPS, the fluorescence intensity was significantly weaker than that of the FITC-LPS group. When ECH was incubated with FITC-LPS, the fluorescence intensity was close to that of the resatorvid + FITC-LPS group and was significantly weaker than that of the FITC-LPS group, which suggests that ECH could competitively bind to the LPS receptor on the cell membrane surface.

### 3.4 Animal experiment results

#### 3.4.1 ECH ameliorates LPS-induced liver histopathological damage in mice

**FIGURE 13:**
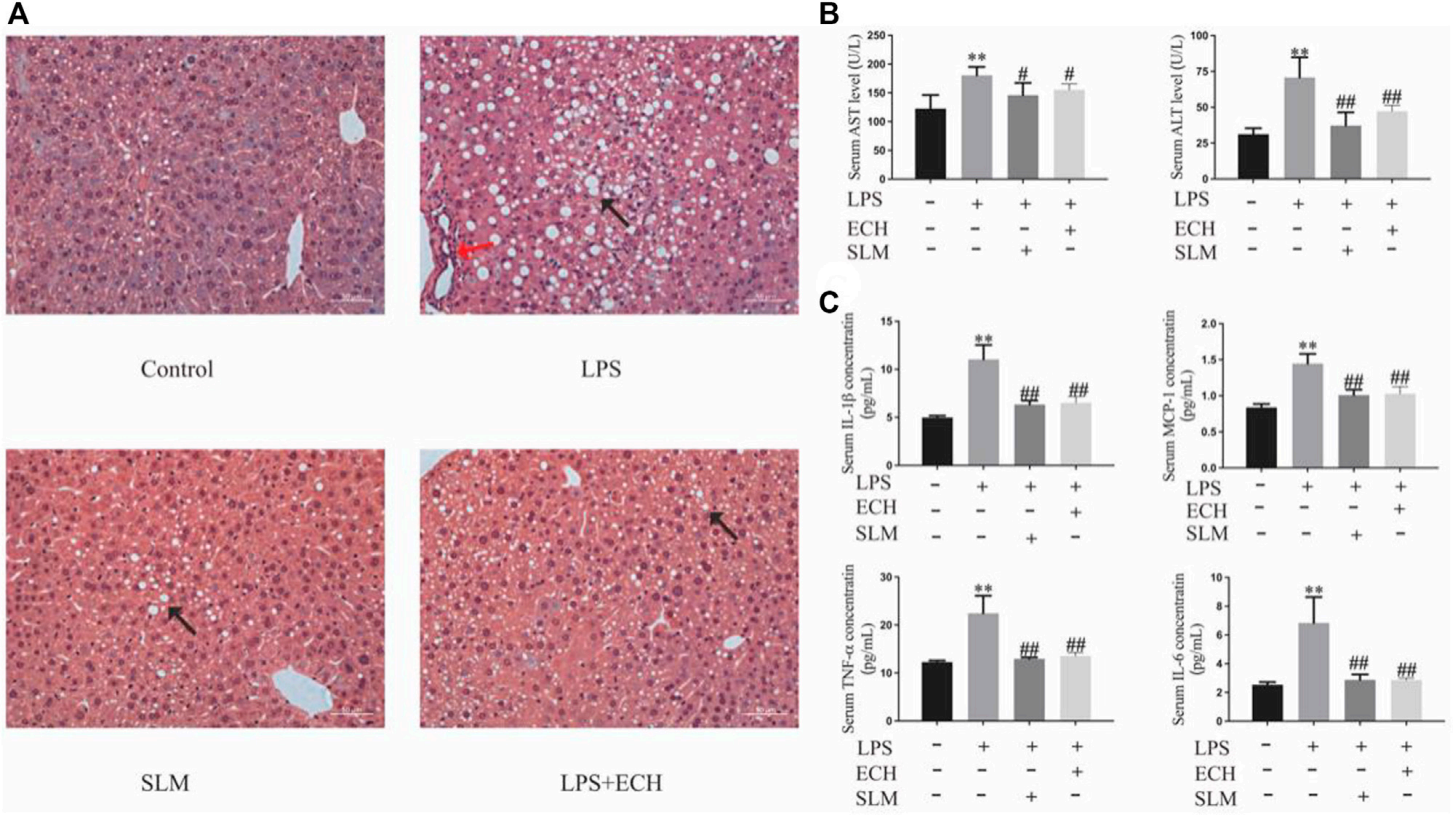
Effects of ECH on LPS-induced liver injury. **(A)** H&E staining of liver tissue in each group; **(B)** ALT and AST analysis of the serum; **(C)** serum levels of the inflammatory factors TNF-α, IL-6, IL-1β, and MCP-1. Compared with the control group, *p < 0.05 and **p < 0.01; in comparison with the LPS group, ^#^P＜0.05 and ##P＜0.01; all data are presented as mean ± SD (*n*=4–6/group).

To assess the effect of ECH treatment on liver tissue, H&E staining was carried out, which is very straightforward and objective. Compared with the control group, the LPS group developed obvious characteristics of liver injury, abnormal hepatic lobule structure, and infiltration of peripheral inflammatory. The aforementioned symptoms were effectively relieved after ECH administration (Figure 13A).

#### 3.4.2 ECH significantly decreases the serum ALT and AST levels in mice induced by LPS

According to the changes in the levels of biochemical enzymes in serum, ALT and AST levels in the LPS group were significantly higher than those in the control group (*p* < 0.01), while both enzyme levels in the ECH groups were decreased with a significant statistical difference (*p* < 0.01) (Figure 13B).

#### 3.4.3 ECH decreases secretion of TNF-α, IL-6, IL-1β, and MCP-1 in mice induced by LPS

To assess the effect of ECH pretreatment on the inflammatory response, the serum secretion levels of pro-inflammatory cytokines TNF-α, IL-6, IL-1β, and MCP-1 were detected by ELISA. As shown in Figure 13C, the levels of TNF-α, IL-6, IL-1β, and MCP-1 in serum were significantly upregulated in the LPS group compared with those in the control group (*p* < 0.01), but the ECH group markedly downregulated the secretion of these pro-inflammatory cytokines (*p* < 0.01).

## 4 Discussion

The inflammatory response is present throughout NAFLD, ranging from nonalcoholic fatty liver to nonalcoholic steatohepatitis (NASH). The inflammatory response has been identified as one of the pathophysiological bases of NAFLD in several studies. Natural products or Chinese herbal medicine are valuable resources for NALFD treatment. Network analysis is an efficient strategy to predict the potential targets of natural products or Chinese herbal medicine (Friedman et al., 2018; Fang et al., 2019; Carpino et al., 2020; Powell et al., 2021). Therefore, the aim of this study was to investigate the hepatoprotective effects of *CD* with network analysis, as well as the *in vitro* and *in vivo* experiments were used to further confirm the results.

First, PhGs have been shown to have good hepatoprotective and immunomodulatory activities (Tian et al., 2021). With the help of the UPLC-Q-TOF/MS/MS technology, we identified the chemical constituents of PhGs in *CD* and further studied their effective components and action mechanism by network analysis. To our knowledge, this is the first report on the integrative strategy of neutral loss and diagnostic ions to identify a total of 34 PhGs from *CD*. Among them, four compounds (compounds 1, 6, 13, and 15) have not been previously reported in *CD*, providing a guide for the identification of other plants of the same genus. Since the PhGs with the same core structure showed the same fragmentation pathway and characteristic fragment ions, we analyzed the fragmentation pathway of typical PhG standards and used the characteristic fragment ions of the standards as diagnostic ions to facilitate the resolution of homologous unknown compounds. Our study not only presented a feasible and generally applicable strategy for the identification of active ingredients in *CD* and other herbal medicines but also laid the foundation for further development and application of PhGs.

Next, we used the 34 components of PhGs identified by UPLC-Q/TOF-MS/MS analysis as candidates for network analysis and a total of 17 active components by database screening for further network analysis. The results of the topological analysis showed that ECH achieved the most targets and that these components were highly correlated with the core targets predicted by NAFLD. Importantly, the levels of nine compounds in the *CD* extract were determined, with ECH having the highest level of 13.45 mg/g. In addition, ECH, as one of the main active components of PhGs, has been well-documented in hepatoprotective studies. For example, Thida et al. (2021) *.* found that ECH attenuated acetaminophen-induced liver injury by reducing oxidative stress and inflammatory cytokines in mice. Li et al. (2014) found that ECH ameliorated d-galactosamine+LPS-induced acute liver injury in mice by inhibiting apoptosis and inflammation (Pei et al., 2018; Thida et al., 2021). Therefore, we infer that ECH is the most likely chemical component to play a key role in hepatoprotection. PPI network topology analysis revealed the key inflammation-related targets. TNF, IL-6, RELA, IL-1β, and TLR4 are important components in the effect of PhGs on NAFLD. Previous researchers had indicated that inflammatory factors such as IL-6 and TNF-α are associated in the development of NAFLD and critical to reducing the inflammatory response for NAFLD prevention and treatment (Jiang et al., 2016; Hou et al., 2021; Tarantino et al., 2021). Pathway and functional enrichment analyses also suggested that PhGs can exert hepatoprotective effects through modulation of LPS responses and TLR signaling-related pathways. In addition, previous studies had shown that LPS initiated intracellular signaling through binding to cell membrane surface receptors and activated TLR4 to induce downstream inflammatory cytokines (Yuan et al., 2018a; Liu et al., 2018b). Of these, TLR4/NF-κB has been shown to play an important role in liver disease (Henao-Mejia et al., 2012; Miura and Ohnishi, 2014; Carpino et al., 2020; Hu et al., 2020). Therefore, we further used cellular assays to confirm that ECH exerts hepatoprotective effects by inhibiting TLR4/NF-κB activation and attenuating the inflammatory response.

The ELISA, RT-qPCR, and Western blot were used to detect inflammatory factors, and the results showed that both 50 and 100 μM of ECH significantly reduced the expression of TNF-α and IL-6. Tao et al. (2021) found that 25–100 μM of ECH was effective in reducing ethanol-induced lipid accumulation and oxidative damage in HepG2 cells. Ye et al. (2019) found that 20–100 μg/ml of ECH had inhibitory proliferative and pro-apoptotic effects on hepatocellular carcinoma HepG2 cells (Ye et al., 2019; Tao et al., 2021), corresponding to the range of concentrations in this study. Substantial evidence has shown that TLR4 is closely associated with NAFLD (Miura and Ohnishi, 2014; Hu et al., 2020). After RT-qPCR and Western blot analysis, ECH was found to reduce the mRNA and protein level expression of TLR4. The MyD88-dependent TLR4 signaling pathway was activated during NAFLD, leading to rapid activation of NF-κB and the generation of pro-inflammatory mediators such as TNF-α, IL-1β, and IL-6 (Lin et al., 2019; Wang et al., 2020c; Feng et al., 2020; Nishimura et al., 2021; Ye et al., 2021). Reduced levels of TLR4, MyD88, and p-P65 expression were linked to lower levels of pro-inflammatory cytokine production, based on the previous study (Li et al., 2020). In the current study, we observed that a reduction in MyD88, NF-κB, p-P65, and TNF-α expression levels was accompanied by a decrease in inflammatory synthesis after ECH administration treatment. These results suggested that ECH may play a hepatoprotective role by inhibiting the activation of TLR4/NF-κB and alleviating the inflammatory response. These findings demonstrate the protective effect of ECH and its potential to develop hepatoprotective drugs.

ALT and AST are the core biochemical indicators commonly used in the clinical evaluation of liver injury (Yuan et al., 2018b). The serum ALT and AST levels in the mice of the LPS group were significantly higher than those in mice of the control group. ECH significantly reduced the aforementioned biochemical indexes, indicating that LPS successfully established a liver damage model, and ECH has a hepatoprotective effect. H&E pathological sections showed that ECH effectively alleviated liver injury in mice. Pro-inflammatory cytokines are direct indicators reflecting the degree of inflammation (Shah et al., 2018). TNF-α, IL-1β, and IL-6 are typical representatives of multifunctional cytokines, playing an important role in the pathogenesis of acute inflammatory response and liver injury (Liu et al., 2018a). After ECH administration intervention, we found that the secretion of TNF-α, IL-1β, MCP-1, and IL-6 inflammatory factors in the serum of mice was significantly reduced. The *in vivo* experimental index detection showed that ECH could effectively reduce the inflammatory response caused by LPS. However, its specific mechanism has not been elucidated, and we will further study the mechanism of TLR4 and other pathways in the future.

## 5 Conclusion

A total of 34 PhGs were identified by UPLC-Q/TOF-MS/MS, and we explored the potential hepatoprotective effects of 17 active ingredients by network analysis. Then, the *in vitro* study with the L02 cell model suggested the anti-inflammatory activities and hepatoprotective effects of PhGs. Further *in vitro* experiments found that PhGs had the effect of reducing the NO level, showing a potential anti-inflammatory effect. As the main active component of PhGs, ECH scored highest in network analysis and molecular docking studies. Therefore, we investigated its anti-inflammatory effects through molecular biology experiments. ECH could reduce the expression of inflammatory factors TNF-α and IL-6, as well as decrease LPS-induced *TLR4*, *TNF-α*, and *IL-6* mRNA levels, and inhibit TLR4, MyD88, p-P65, and TNF-α protein expression. These experiments also preliminarily verified that it achieves anti-inflammatory effects through the TLR4/NF-κB signaling pathway. The target competition assay showed that ECH and LPS competitively bound TLR4. Finally, we found that ECH had a hepatoprotective effect on LPS-induced liver injury mice through *in vivo* animal experiments. To sum up, PhGs can be used as anti-inflammatory active components of *CD*, and they play a hepatoprotective role by reducing the inflammatory response. Altogether, the results of this study both explored the anti-inflammatory effect of ECH and provided a novel understanding of the active ingredients and mechanisms of action of herbal medicines. In the long run, it allows the combination of systematic pharmacology with qualitative and quantitative analyses to better grasp the mechanism of herb medicines.

## Data Availability

The datasets presented in this study can be found in online repositories. The names of the repository/repositories and accession number(s) can be found in the article/Supplementary Material.
