# Supplementary material for: Potential hepatoprotective effects of Cistanche deserticola Y.C. Ma: Integrated phytochemical analysis using UPLC-Q-TOF-MS/MS, target network analysis, and experimental assessment
Source: Front Pharmacol. 2022 Oct 12;13:1018572. doi: 10.3389/fphar.2022.1018572 (PMC9597371; doi:10.3389/fphar.2022.1018572)
Supplement: Supplementary file 1 [file DataSheet1.docx]

Supplementary Material

**Table S1.** The structure information of standard solution

| **No.** | **Compounds** | **Chemical Structure** | **R1** | **R2** | **R3** | **R4** | **R5** | **Mfg.ID** |
| --- | --- | --- | --- | --- | --- | --- | --- | --- |
| 1 | 2′-acetylacteoside |  | Ac | Rha | Cf | H | OH | MUST-17031504 |
| 2 | acteoside |  | H | Rha | Cf | H | OH | MUST-17022620 |
| 3 | echinacoside |  | H | Rha | Cf | Glc | OH | MUST-17030701 |
| 4 | isoacteoside |  | H | Rha | H | Cf | OH | MUST-17031514 |
| 5 | osmanthuside B |  | H | Rha | Cm | H | H | CTR-5360 |
| 6 | poliumoside |  | H | Rha | Cf | Rha | OH | CTR-3882 |
| 7 | salidroside |  | H | H | H | H | H | MUST-17031511 |
| 8 | tubuloside A |  | Ac | Rha | Cf | Glc | OH | MUST-17031504 |
| 9 | tubuloside B |  | Ac | Rha | H | Cf | OH | CHB-G-036 |
| 10 | cistanoside A |  | H | Rha | Cf | Glc | OH | CTR-3791 |

Maker: Ac: acetyl; Cf: trans-caffeoyl; Cm: trans-p-coumaroyl; Glc: β-D-glucopyranose; Rha: α-L-rhamnopyranose

**Table S2.** The detailed information of antibodies

| **No.** | **Name** | **Production Manufacturer** | **Mfg.ID** |
| --- | --- | --- | --- |
| 1 | NF-κB | Cell Signaling Technology | #8242 |
| 2 | TNF-α | ABcam | ab667 |
| 3 | MyD88 | ABclonal, | A0980 |
| 4 | Resatorvid | MedChemExpress | HY-11109 |
| 5 | β-Actin Rabbit mAb | Cell Signaling Technology, Inc | #4970s |
| 6 | TLR4 | Santa Cruz Biotechnology, Inc | sc-293072 |
| 7 | Anti-rabbit lgG HRP-linked | Cell Signaling Technology,Inc | #7074 |
| 8 | Antibody Phospho-NF-κB p65 primary antibodies | Cell Signaling Technology | 3033S |
| 9 | Goat anti-Mouse lgG (H+L) Cross-Adsorbed Secondary Antibody,HRP | Thermo Fisher Scientific, | Cat# G-21040 |

**Table S3.** Gradient elution program.

| **Time/min** | **Mobile phase A/%** |
| --- | --- |
| 0-8 | 2-12 |
| 8-16 | 12-16 |
| 16-19 | 16 |
| 19-22 | 16-19 |
| 22-25 | 19 |
| 25-30 | 19-25 |
| 30-32 | 25 |
| 32-35 | 25-30 |
| 35-38 | 30-40 |
| 38-40 | 40-50 |

**Table S4.** The information of target gene and internal reference gene

| **No.** | **Name** | **Sequence (5’-3’)** | **Size** |
| --- | --- | --- | --- |
| 1 | *β-ACTB-F* | 5’-TGGCACCCAGCACAATGAA-3’ | 19 |
| 2 | *β-ACTB-R* | 5’-CTAAGTCATAGTCCGCCTAGAAGCA-3’ | 25 |
| 3 | *IL-6-F* | 5’-AAGCCAGAGCTGTGCAGATGAGTA-3’ | 24 |
| 4 | *IL-6-R* | 5’-TGTCCTGCAGCCACTGGTTC-3’ | 20 |
| 5 | *TNF-α-F* | 5’-CTGCCTGCTGCACTTTGGAG-3’ | 20 |
| 6 | *TNF-α-R* | 5’-ACATGGGCTACAGGCTTGTCACT-3’ | 23 |
| 7 | *TLR4-F* | 5’-TGGCCCTAAACCACACAGAA-3’ | 20 |
| 8 | *TLR4-R* | 5’-TGGCCCTAAACCACACAGAA-3’ | 20 |

**Table S5.** Molecular docking of 15 components to TLR4 (5uc9)

| **No.** | **Name** | **Molecular formula** | **Molecular weight** | **Center coordinates (x, y, z)** | **Box size (x, y, z)** | **Binding energies (kJ/mol)** |
| --- | --- | --- | --- | --- | --- | --- |
| 1 | salidroside | C_14_H_19_O_7_ | 299.1133 | 18.81, 18.107, -5.684 | 54.46, 59.15, 50.7 | -7.5 |
| 2 | cistanoside F | C_21_H_27_O_13_ | 487.1456 | 18.81, 18.107, -5.684 | 52.32, 64.48, 58.4 | -6.6 |
| 3 | cistanoside E | C_21_H_31_O_12_ | 475.1813 | 18.81, 18.107, -5.684 | 50.4, 59.85, 46.2 | -7.2 |
| 4 | echinacoside | C_35_H_45_O_20_ | 785.2492 | 18.81, 18.107, -5.684 | 48.9, 57.05, 46.18 | -8.6 |
| 5 | cistanoside A | C_36_H_47_O_20_ | 799.2674 | 18.81, 18.107, -5.684 | 53.08, 57.41, 47.67 | -8.4 |
| 6 | acteoside | C_29_H_35_O_15_ | 623.1987 | 18.81, 18.107, -5.684 | 57.63, 62.18, 47.02 | -8.3 |
| 7 | cistanoside B | C_37_H_49_O_20_ | 813.2802 | 18.81, 18.107, -5.684 | 51.03, 58.45, 46.39 | -7.6 |
| 8 | cistanoside D | C_31_H_39_O_15_ | 651.2292 | 18.81, 18.107, -5.684 | 61.42, 55.83, 49.13 | -6.9 |
| 9 | tubuloside A | C_37_H_47_O_21_ | 827.2615 | 18.81, 18.107, -5.684 | 48.58, 57.75, 51.33 | -7.6 |
| 10 | tubuloside B | C_31_H_37_O_16_ | 665.2067 | 18.81, 18.107, -5.684 | 56.55, 58.0, 53.65 | -7.9 |
| 11 | osmanthuside B | C_29_H_35_O_13_ | 591.2087 | 18.81, 18.107, -5.684 | 61.07, 63.61, 50.89 | -8.3 |
| 12 | decaffeoylacteoside | C_20_H_29_O_12_ | 461.1662 | 18.81, 18.107, -5.684 | 55.66, 59.54, 50.48 | -6.6 |
| 13 | cistanoside C | C_30_H_37_O_15_ | 637.2129 | 18.81, 18.107, -5.684 | 56.99, 58.23, 56.99 | -8.0 |
| 14 | 2’-acetylacteoside | C_31_H_37_O_16_ | 665.2087 | 18.81, 18.107, -5.684 | 50.7, 57.27, 55.39 | -5.6 |
| 15 | plantainoside C or isomer | C_30_H_37_O_15_ | 637.2130 | 18.81, 18.107, -5.684 | 54.43, 65.08, 63.9 | -7.6 |
| 16 | isosyringalide-3’-α-L-rhamnopyranoside or Isomer | C_29_H_35_O_14_ | 607.2015 | — | — | — |
| 17 | syringalide A-3’-α-L-rhamnopyranoside | C_29_H_35_O_14_ | 607.2036 | — | — | — |

**
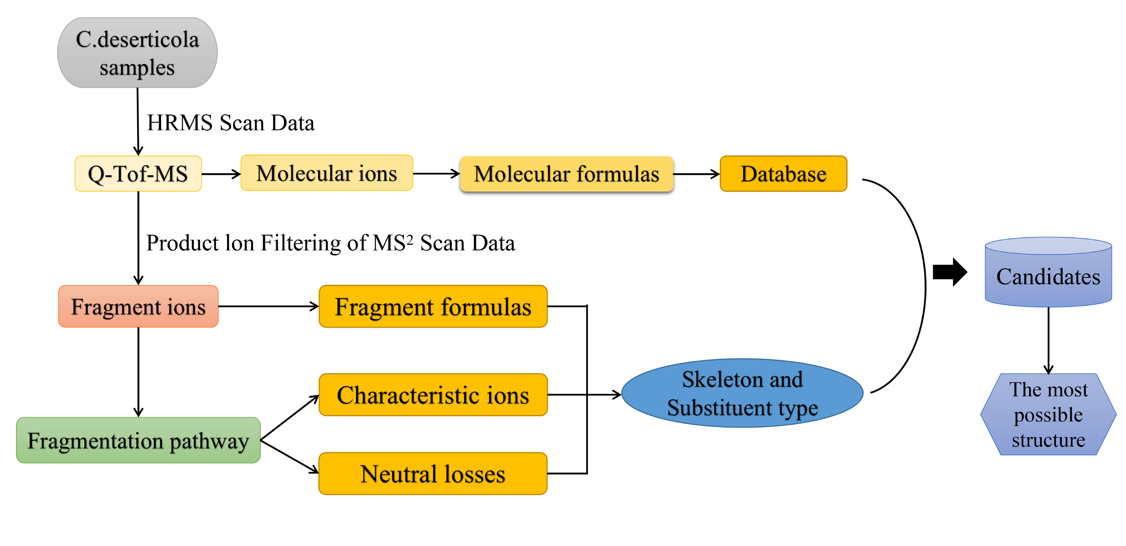
**

**Supplementary Figure 1.** The identification process of PhGs in the 70% ethanol extract of *CD*.


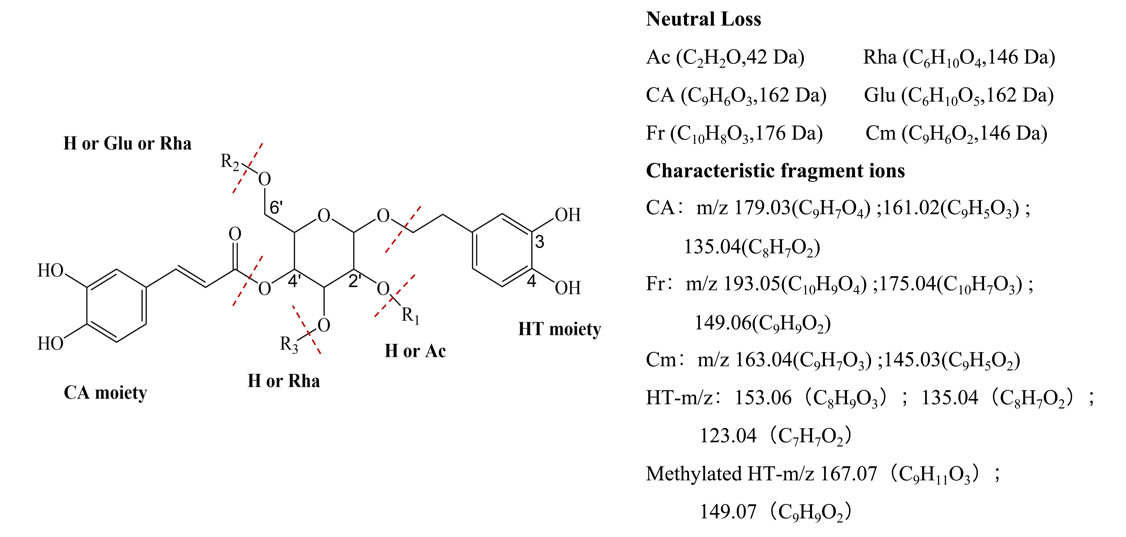


**Supplementary Figure 2.** The structures and fragmentation pathway of PhGs.


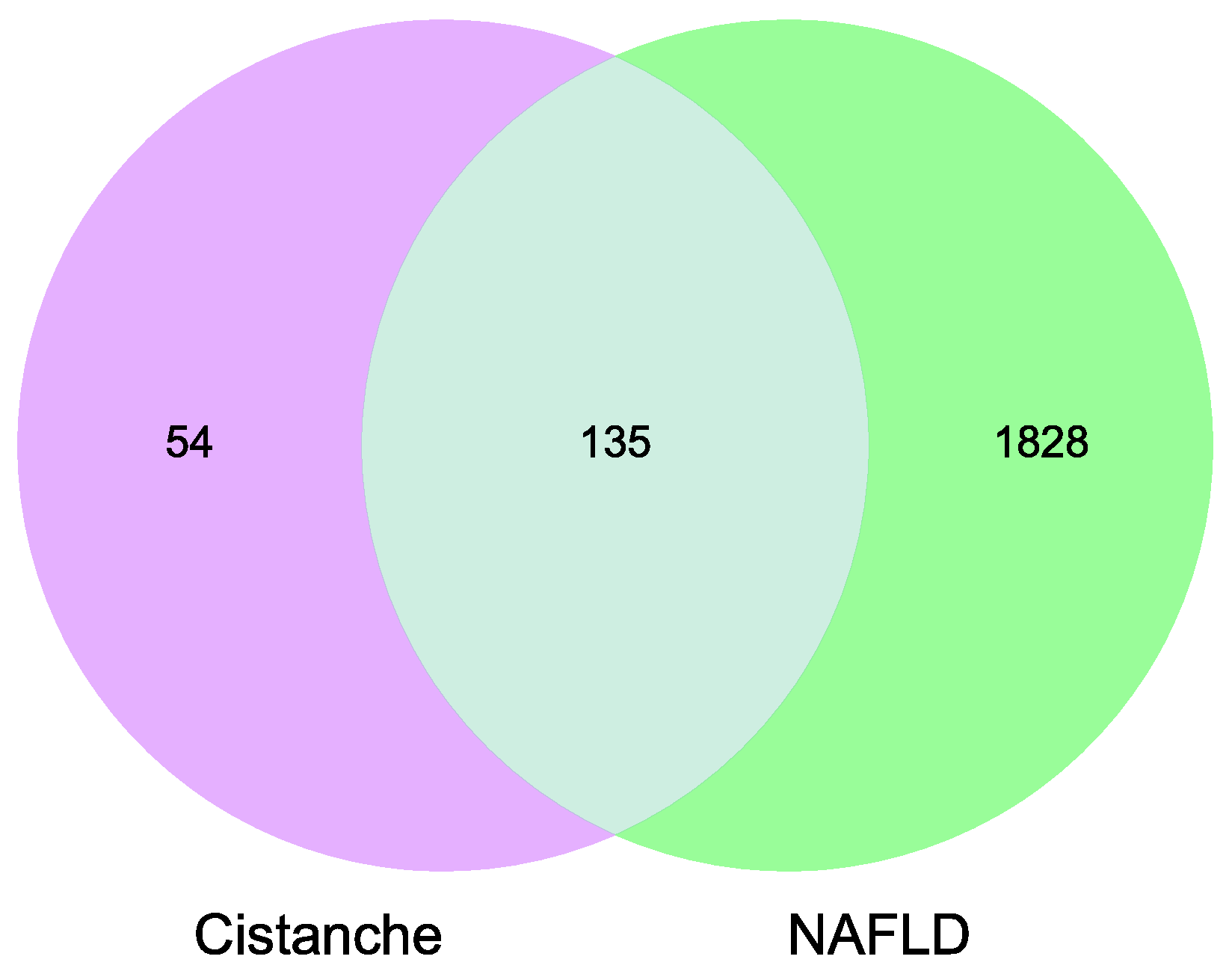


**Supplementary Figure 3.** The venn diagram of predicted targets of *CD* and NAFLD;


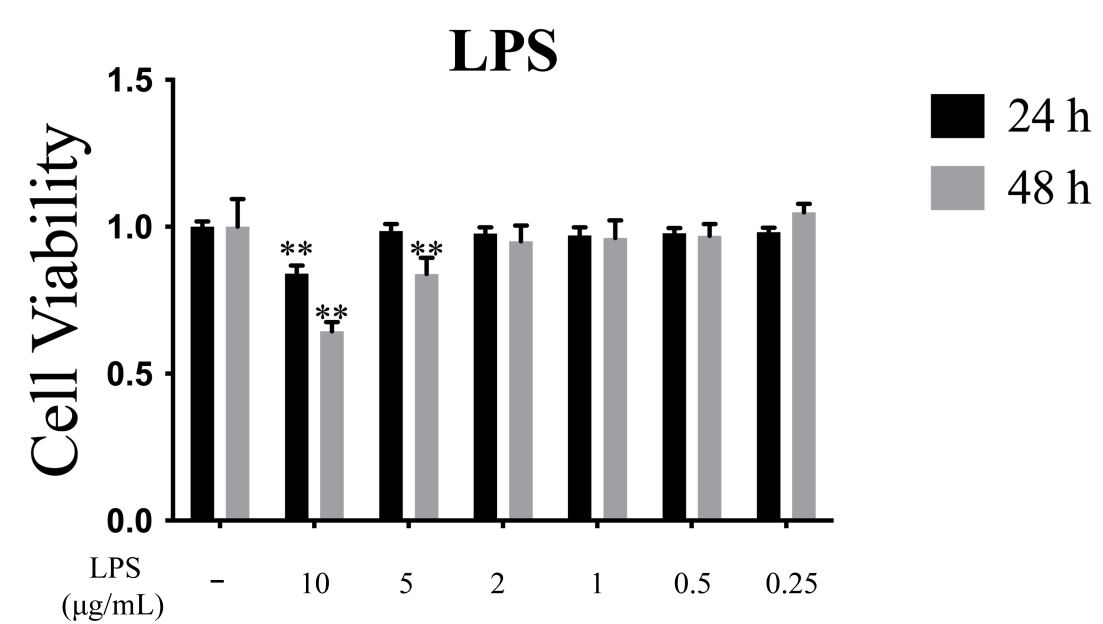


**Supplementary Figure 4.** Effects of different doses and durations of LPS on L02 cell viability (n > 3).

1. **Other compound fragmentation patterns of PhGs**

The quasi-molecular ions of both peak 13 and peak 14 (C_36_H_47_O_20_, eluted at 15.01 and 14.82 min, respectively) are m/z 799.27 [M-H]^-^, which is 14 Da (i.e. CH_2_) more than peak 10 (m/z 785.25, C_35_H_45_O_20_). In the MS^2^ cleavage, the fragment ion m/z 785.25 (C_35_H_45_O_20_) attributed to loss the corresponding CH_2_. The loss of one molecule of caffeoyl (C_9_H_6_O_3_, -162 Da) from the quasi-molecular ion in the MS^2^ spectrum forms a fragment ion m/z 637.24 at peak 14 (C_27_H_41_O_17_), which further losses a neutral molecule of rhamnose (C_6_H_10_O_4_, 146 Da) and yields the corresponding fragment ions m/z 491.18 (C_21_H_31_O_13_). These fragment ions are still more CH_2_ than the corresponding fragment ions m/z 623.22 (C_26_H_39_O_17_) and 477.16 (C_20_H_29_O_13_) at peak 10, and the characteristic fragmentation ions m/z 179.03, 161.02 and 135.04 associated with the caffeoyl structure were observed. The fragment ions m/z 167.07 (C_9_H_11_O_3_) and dehydration peak m/z 149.07 (C_9_H_9_O_2_) suggested that the hydroxyl group on the PhGs was methylated, but this compound was not found in the database by matching the characteristics, and then, it was attributed to unknown. The loss of one molecule of methylated caffeoyl (C_10_H_8_O_3_, -176 Da) from the quasi-molecular ion in the MS^2^ spectrum forms a fragment ion m/z 623.22 (C_26_H_39_O_17_) at peak 13, which further losses neutral molecules of rhamnose (C_6_H_10_O_4_, 146 Da) and glucose (C_6_H_10_O_5_, 162 Da ), and the same fragment ions at m/z 477.16 and 461.17 are produced in the peak 10. The characteristic fragmentation ions at m/z 153.06 and 123.04 related to the structure of PhGs can be found. The fragment ions m/z 193.05 (C_10_H_9_O_4_) and 175.04 (C_10_H_7_O_3_) are more CH_2_ than the characteristic fragment ions m/z 179.03 and 161.02. The hydroxyl group on caffeoyl was presumed to be methylated. After searching the compound library and comparing with the literatures, it was identified as cistanoside A.

Peaks 11, 12, and 20 (C_35_H_45_O_19_, eluted at 14.49, 14.14 and 19.96 min, respectively) exhibit the same quasi-molecular ion [M-H]^-^ m/z 769.25 in the negative ion mode. In the MS^2^ spectrum, there are some differences between the above peaks. In the peak 11, the loss of one molecule of caffeoyl (C_9_H_6_O_3_, -162 Da) from the quasi-molecular ion produces a fragment ion m/z 607.22 (C_26_H_39_O_16_), which further losses the molecules of rhamnose (C_6_H_10_O_4_, 146 Da) and glucose (C_6_H_10_O_5_, 162 Da ), and then produces the corresponding fragment ions m/z 461.17 ( C_20_H_29_O_12_) and 445.17 (C_20_H_29_O_11_). The characteristic fragmentation ions m/z 179.03, 161.02 and 135.04 related to the caffeoyl structure were observed. Peak 12 produces fragment ions m/z 623.22 (C_26_H_39_O_17_), which differs from the excimer ion peak (C_9_H_6_O_2_, -146Da). The fragment ions m/z 163.04 (C_9_H_7_O_3_) and 145.03 (C_9_H_5_O_2_) are less one O than the characteristic fragment ions m/z 179.03 and 161.02 of caffeoyl, presuming that peak 12 is a coumaryl group with one hydroxyl group removed from the caffeoyl group. The fragment ion further losses the molecules of rhamnose (C_6_H_10_O_4_, 146 Da) and glucose (C_6_H_10_O_5_, 162 Da), yielding the corresponding fragment ions m/z 477.16 (C_20_H_29_O_13_) and m/z 461.17 (C_20_H_29_O_12_). The characteristic fragment ions m/z 153.06 and 123.04 associated with the structure of the PhGs were observed. In the peak 20， the quasi-molecular ion losses one molecule of rhamnose (C_6_H_10_O_4_, -146 Da) and produces a fragment ion m/z 607.20 (C_29_H_35_O_15_), which further losses one molecule of caffeoyl (C_9_H_6_O_3_, -162 Da) and produces the fragment ion m/z 461.17 (C_20_H_29_O_12_). The characteristic fragment ions m/z 179.03, 161.02 and 135.04 associated with the caffeoyl structure were visible, as well as the characteristic fragmentation peaks m/z 153.06 and 123.04 associated with the PhGs structure were observed. Compared with the reference compound of poliumoside, it is consistent with peak 20. Peaks 11 and 12 were identified as cistantubuloside A and cistantubuloside B1/B2 respectively based on the distinction of structurally related characteristic ion peaks.

Peaks 21 and 22 or 26 (m/z 607.20, C_29_H_35_O_14_, eluted at 20.21 and 20.82 or 23.16 min, respectively), with quasi-molecular ion [M-H]^-^ m/z 607.20 in the negative ion mode. In the MS^2^ spectrum, there are some differences between them. Peak 21, with the loss of one molecule of caffeoyl (C_9_H_6_O_3_, -162 Da), yields fragment ion m/z 445.17 (C_20_H_29_O_11_), and characteristic fragment ions m/z 179.03, 161.02 and 135.04 associated with the caffeoyl structure were observed. Peak 22 or 26 yield fragment ion m/z 461.16 (C_20_H_29_O_12_), which differs from the quasi-molecular ion m/z 146 (C_9_H_6_O_2_). Peaks 22 or 26 are presumed to be coumaroyl groups with one hydroxyl group removed from caffeoyl by producing fragment ions m/z 163.04 (C_9_H_7_O_3_) and 145.03 (C_9_H_5_O_2_), which have one O less than the characteristic fragment ions m/z 179.03 and 161.02 of caffeoyl. The characteristic fragment ions m/z 153.06 and 123.04 related to the structure of PhGs were observed. Peaks 21 and 22 or 26 were identified as syringalide A-3-α-L-rhamnopyranoside and isosyringalide-3-α-L-rhamnopyranoside or isomers respectively based on the distinction of their structurally related characteristic ionic peaks.

In the negative ion mode, peaks 23 and 24 or 27 (m/z 637.21, C_30_H_37_O_15_, eluted at 21.65 and 21.93 or 23.39 min, respectively) possess the same quasi-molecular ion [M-H]^-^ m/z 637.21, which has 14 Da (i.e. CH_2_) more than peaks 16 and 19 (m/z 623.20, C_29_H_35_O_15_). The fragment ion m/z 623.20 (C_29_H_35_O_15_) was formed by the loss of the corresponding CH in both MS^2^ cleavages. The quasi-molecular ion in the MS^2^ spectrum forms a fragment ion m/z 475.18 (C_21_H_31_O_12_) in the peak 23 by the loss of caffeoyl (C_9_H_6_O_3_, -162 Da), which further losses a molecule of rhamnose (C_6_H_10_O_4_, 146 Da) and yields the corresponding fragment ion m/z 329.12 (C_15_H_21_O_8_). The above key ions have one CH_2_ more than the corresponding fragment ions m/z 461.17 and 315.11 in the peaks of 16and 19. The characteristic fragment ions m/z 179.03, 161.02 and 135.04 for the caffeoyl structure-related peaks were observed. The hydroxyl group on the PhGs was presumed to be methylated through producing the fragment ion m/z 167.07 (C_9_H_11_O_3_) and dehydration peak 149.07 (C_9_H_9_O_2_). After searching the compound library and comparing with literatures, it was identified as cistanoside C^[24]^. The quasi-molecular ion in the MS^2^ spectrum forms the fragment ion m/z 461.17 (C_20_H_29_O_12_ )in the peaks of 24 or 27 by the loss of one molecule of methylated caffeoyl (C10H8O3, -176 Da), which further losses a molecule of rhamnose (C_6_H_10_O_4_, 146 Da) and yields the same fragment ion m/z 461.17 in the peaks of 16and 19. The characteristic fragment ions m/z 153.06 and 123.04 related to the structure of PhGs were observed. The hydroxyl group on the caffeoyl group was presumed to be methylated according to the fragment ions m/z 193.05 (C_10_H_9_O_4_) and 175.04 (C_10_H_7_O_3_) with more CH_2_ than the characteristic fragment ions m/z 179.03 and 161.02 produced by the caffeoyl group. After searching the compound library and comparing with literatures, the component was identified as plantainoside C or its isomer.

Peaks 29 (24.15 min, m/z 591.21, C_29_H_35_O_13_) and 32 (27.02 min, 649.21, C_31_H_37_O_15_) can produce the characteristic fragment ions m/z 163.04 (C_9_H_7_O_3_) and 145.03 (C_9_H_5_O_2_) corresponding to the removal of a hydroxyl group of coumaroyl from caffeoyl. The quasi-molecular ion in the MS^2^ spectrum forms the fragment ion m/z 445.17 (C_20_H_29_O_11_) in the peak of 29 by the loss of one molecule of coumaroyl (C_9_H_6_O_2_, -146 Da). After comparing with the reference compound of osmanthuside B, it is consistent with peak 29. In the peak 32, the fragment ions m/z 607.20 (C_29_H_35_O_14_) and 503.18 (C_22_H_31_O_13_) are produced by the loss of acety (C_2_H_2_O, -42 Da) and coumaroyl (C_9_H_6_O_2_, -146 Da). After searching the compound library and comparing with literatures, the compound was identified as salsaside F.

Peak 4 has an earlier retention time, which 136 Da less than the acteosid and isoacteosid (m/z 623.20, C_29_H_35_O_15_), i.e. a PhGs structure (C_8_H_8_O_2_). The characteristic fragment ions at m/z 179.03, 161.02 and 135.04 related to the caffeoyl structure are visible in the MS^2^ spectrum. Compared with the retention time and mass spectra information of the reference compound of cistanoside F, it is consistent with peak 4.
